# Microparticles mediated cross-talk between tumoral and endothelial cells promote the constitution of a pro-metastatic vascular niche through Arf6 up regulation

**DOI:** 10.1007/s12307-013-0142-2

**Published:** 2014-01-15

**Authors:** Jennifer Pasquier, Hamda Al Thawadi, Pegah Ghiabi, Nadine Abu-Kaoud, Mahtab Maleki, Bella S. Guerrouahen, Fabien Vidal, Bettina Courderc, Gwenael Ferron, Alejandra Martinez, Haya Al Sulaiti, Renuka Gupta, Shahin Rafii, Arash Rafii

**Affiliations:** 1 Stem Cell and Microenvironment Laboratory, Weill Cornell Medical College in Qatar, Education City, Qatar Foundation, Doha, Qatar; 2 EA 4553, Institut Claudius Regaud, Toulouse, France; 3 Department of Genetic Medicine, Weill Cornell Medical College, New York, NY USA; 4 Department of Surgery, Institut Claudius Regaud, Toulouse, France; 5 Department of Advanced Gynecologic Surgery, Hospital Arnaud de Villeneuve, CHUR, Montpellier, France; 6 Department of Genetic Medicine and Obstetrics and Gynecology, Weill Cornell Medical College, Stem Cell and Microenvironment Laboratory Weill Cornell Medical College in Qatar, Qatar-Foundation, PO: 24144 Doha, Qatar

**Keywords:** Cancer, Tumor microenvironment, Cell-cell interactions, Metastasis, Microparticles, Endothelial cells

## Abstract

The tumor stroma plays an essential role in tumor growth, resistance to therapy and occurrence of metastatic phenotype. Tumor vessels have been considered as passive conducts for nutrients but several studies have demonstrated secretion of pro-tumoral factors by endothelial cells. The failure of anti-angiogenic therapies to meet expectations raised by pre-clinical studies prompt us to better study the cross-talk between endothelial and cancer cells. Here, we hypothesized that tumor cells and the endothelium secrete bio-active microparticles (MPs) participating to a functional cross-talk. We characterized the cancer cells MPs, using breast and ovarian cancer cell lines (MCF7, MDA-MB231, SKOV3, OVCAR3 and a primary cell lines, APOCC). Our data show that MPs from mesenchymal-like cell lines (MDA-MB231, SKOV3 and APOCC) were able to promote an activation of endothelial cells through Akt phosphorylation, compared to MPs from epithelial-like cell lines (OVCAR3 and MCF7). The MPs from mesenchymal-like cells contained increased angiogenic molecules including PDGF, IL8 and angiogenin. The endothelial activation was associated to increased Arf6 expression and MPs secretion. Endothelial activation functionalized an MP dependent pro-tumoral vascular niche promoting cancer cells proliferation, invasiveness, stem cell phenotype and chemoresistance. MPs from cancer and endothelial cells displayed phenotypic heterogeneity, and participated to a functional cross-talk where endothelial activation by cancer MPs resulted in increased secretion of EC-MPs sustaining tumor cells. Such cross-talk may play a role in perfusion independent role of the endothelium.

**Electronic supplementary material:**

The online version of this article (doi:10.1007/s12307-013-0142-2) contains supplementary material, which is available to authorized users.

## Introduction

Several mechanisms mediate the cross talk between cancer and stromal cells: (i) paracrine or juxtacrine cytokine/receptor interaction [1,2] (ii) direct cell contact and material exchange [3–5] (iii) vesicles mediated cell communication [6]. While vesicles share some common features they also differ by their morphologic, proteomic or lipidic contents. Despite recent effort to comprehensively classify them, literature on shed microvesicles is still confusing, mainly due to conflicting denominations [7]. Most studies have focused on exosomes. These 50 to 100 nm diameter vesicles are generated by a budding of the endosomal membrane producing multivesicular bodies (MVB) and are released in the extracellular matrix upon MVB fusion with the plasma membrane [8,9]. Cells also shed a heterogeneous population of vesicles different from both exosomes and apoptotic bodies. These vesicles are larger, ranging from 100 nm to few micrometers in diameter, and are described as shedding vesicles, oncosomes, microvesicles, microparticles, ectosomes, membrane particles or exosomes-like vesicles [10]. In our study, we will refer to these structures as microparticles (MPs).

MPs mediate multiple functions through local and systemic shuttling of proteins, mRNAs or miRNAs [7,11,12]. Tumor derived MPs have been implicated in mechanisms such as: transfer of tumor antigens to dendritic cells [13], or acquisition of resistance [3,14,15]. They also play a role in cross-talk with ECs [16,17] or bone marrow cells [6]. In the neoplastic setting, MPs facilitate extracellular matrix invasion and immune response [18,19]. They also play a role in the acquisition of chemo-resistance [3,14,15]. All tumor cells could potentially secrete MPs and their concentration might be related to invasiveness and disease progression [20]. Recently, Lyden’s group had shown that melanoma-derived MPs displayed a specific signature and were able to educate bone marrow derived progenitor cells in order to induce a pre-metastatic niche, supporting in return tumor spread and growth [6].

Most of the literature on tumor angiogenesis concentrated on how vessels are recruited and structurally distorted to stimulate tumor progression [21]. However, anti-angiogenic strategies have not met so far the clinical expectations, despite their ability to disrupt tumor vessels proposing a more complex role for the endothelium [22,23]. A more direct role for the endothelial cells in tumor growth and metastasis has been proposed [24,25]. Tumor vessels are not simply passive tubes for nutrients since the perfusion independent properties of endothelial cells (ECs) have been described in the developmental and neoplasic contexts [26–29]. The role of paracrine factors such as angiogenic peptide basic fibroblast growth factor (bFGF) have been illustrated in different tumors including leukemia [30,31], and solid tumors such as IL-6 (interleukin 6) in melanoma [32] or jagged 1 in colon cancer [33]. In this context endothelial cells pro-tumoral role is mediated through both contact-dependent and contact-independent mechanisms. Similarly, we previously described how they modulate cancer cells (CCs) phenotype toward a chemoresistant profile through direct contact and organelle transfer [28].

Two studies have focused on MPs-mediated cross talk between tumors cells and ECs. Svensson et al. have demonstrated that hypoxic cancer cells released substantial amounts of MPs-associated tissue factors, triggering TF/VIIa–dependent activation of hypoxic ECs [17]. Kawamoto et al. illustrated the ability of Tumor derived microvesicles (TMV) to activate the Pi3Kinase/Akt pathway in ECs through active endocytosis [16]. However, the relationship between MPs uptake and its functional consequences has not been elucidated yet, thus it remains unclear how ECs and cancer cells phenotype can be affected by MPs.

Here we hypothesize that tumor and endothelial cells secrete bio-active MPs participating in a functional cross talk. Our data show that MPs from mesenchymal-like metastatic cell lines (MDA-MB231 and SKOV3, a primary cell lines) were able to induce a phosphorylation of Akt in ECs compared to MPs from epithelial-like cell lines (OVCAR3 and MCF7). The Akt activation in ECs increased Arf6 expression and functionalized a MPs dependent vascular niche enhancing tumor cells pro-metastatic properties.

## Results

### Cancer cells lines display epithelial or mesenchymal phenotype

**Fig. 1:**
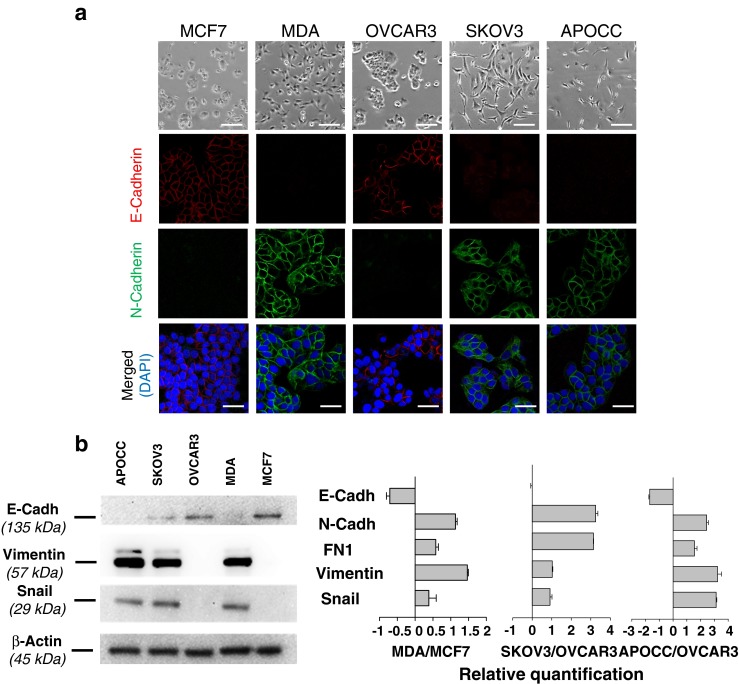

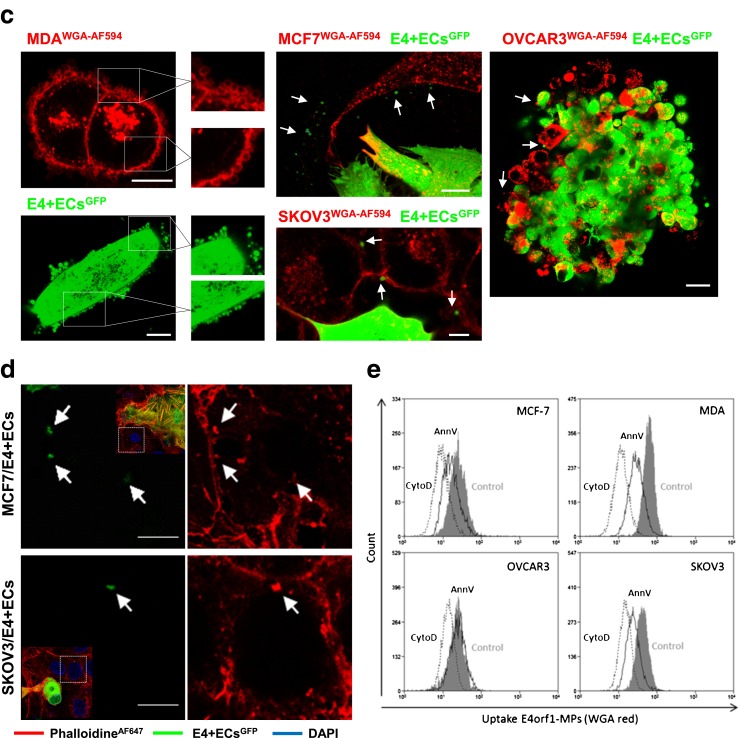
Characterization of mesenchymal and epithelial phenotype. **a** Phase contrast microscopy pictures show significant morphological difference concordant with an epithelial phenotype (MCF7 and OVCAR3) and a mesenchymal phenotype (MDA, SKOV3 and APOCC). Scale bar: 150 μm. Cells were stained for E-cadherin or N-cadherin and analyzed by confocal microscopy. Presence of N-cadherin and E-cadherin staining is concordant with the first observations of the cells body shape. Scale bar 50 μm. **b** Western blot analysis of the different cells lines for mesenchymal (Vimentin and Snail) and epithelial (E-cadherin) markers confirm the difference between MCF7 and OVCAR3 on one part (epithelial phenotype) and SKOV3, MDA and APOCC on the other part (mesenchymal phenotype). MDA, SKOV3 and APOCC expressed mesenchymal genes at levels significantly higher than MCF7 and OVCAR3, as determined by real-time qPCR. Relative transcript levels are represented as the log_10_ of ratios between the 2 subpopulations of their 2^–ΔΔCp^ real-time PCR values. **c** eGFP-E4+ECs were co-cultured with tumor cells for 3 days. Before imaging by confocal microscopy, co-cultures were stained with Alexa Fluor 594 conjugated-wheat germ agglutinin (WGA). GFP-MPs and WGA-MPs are detected floating between cells and on cell membranes. Typical characteristics of MPs are observed: (i) particles smaller than 1 μm, (ii) budding at the membrane. Scale bar: 5 μm. Confocal imaging of MDA/eGFP-E4+ECs sphere. Spheroids of MDA and eGFP-E4+ECs were grown in 3D media for 5 days. MDA cells were stained with Pkh red before spheroids formation. Red or green MPs (*arrow*) are visible inside the sphere. Scale bar: 10 μm. **d** eGFP-E4+ECs were co-cultured with MCF7 or SKOV3 cells for 3 days. Before imaging by confocal microscopy, fixed cells were stained with DAPI and AlexaFluor 647 conjugated-phalloidin. Fixed cells were stained with WGA, DAPI and AlexaFluor 647 conjugated-phalloidin. Arrows demonstrate co-localization of eGFP-E4+ECs-MPs (*green*) and actin patches (*red*). Scale bar 10 μm. **e** MPs from E4+ECs were extracted from 80 % confluent cells and labeled with Alexa Fluor 594 conjugated-wheat germ agglutinin (WGA). Each cancer cells lines were incubated with E4+ECs-MPs for 24 h at 37°c in presence or absence (control) of Annexin V (AnnV) or cytochalsine D (CytoD). MPs uptake quantification was made by flow cytometry. MPs uptake decrease in presence of cytoskeleton heckler or inhibitors

We used 4 different cancer cells lines, 2 breast (MCF7 and MDA-MB231) and 2 ovarian (OVCAR3 and SKOV3), as well as a primary ovarian cancer cell line derived in our laboratory from ascites of a patient with Stage III serous adenocarcinoma (APOCC). All cell lines displayed different morphology (Fig. 1a). MCF7 and OVCAR3 were polygonal in shape with regular dimensions, and grew in discrete patches. MDA, SKOV3 and APOCC had mesenchymal-like features and grew with less interaction. Concordantly MDA, SKOV3 and APOCC expression of mesenchymal markers was confirmed by confocal microscopy (Fig. 1a), western blot and qPCR (Fig. 1b) while OVCAR3 and MCF7 expressed epithelial markers.

### Cancer cells and endothelial cells secrete and uptake microparticles through an active process

To track spontaneous excretion and uptake of MPs, we used eGFP-E4+ECs and stained the cancer cells or HUVECs with WGA [3]. Live cell imaging showed a typical MP release from the different cell types (Fig. 1c, right panel). In 2D and 3D co-culture settings we observed secretion and uptake of MPs by the different cell lines used (Fig. 1c, middle and left panel). To demonstrate an active uptake of MPs we showed the co-localization of actin in areas of MPs uptake on confocal microscopy (Fig. 1d and Supplementary Figure 1). We also used an inhibitor of actin polymerization, Cytochalasin D, and annexin V which is able to bind to membrane phosphatidylserine and thus inhibit the active uptake [34], in both experimental settings MPs uptake was inhibited (Fig. 1e). Finally we showed the absence of MPs uptake at 4 °C compare to 37 °C for all CCs using confocal microscopy, flow cytometry and time-lapse imaging (Supplementary figure 2A) [16]. We noticed aggregation of EC-MPs at cancer cells’ membrane at 4 °C (Supplementary figure 2A, right panels) suggesting adhesion without uptake, which relies on the functionality of the cytoskeleton. Similarly E4+EC uptake of CC-MPs was inhibited at 4 °C and activated at 37 °C (Supplementary figure 2B).

### Mesenchymal-type CCs derived MPs have a different effect on ECs compare to epithelial-type CCs MPs

**Fig. 2:**
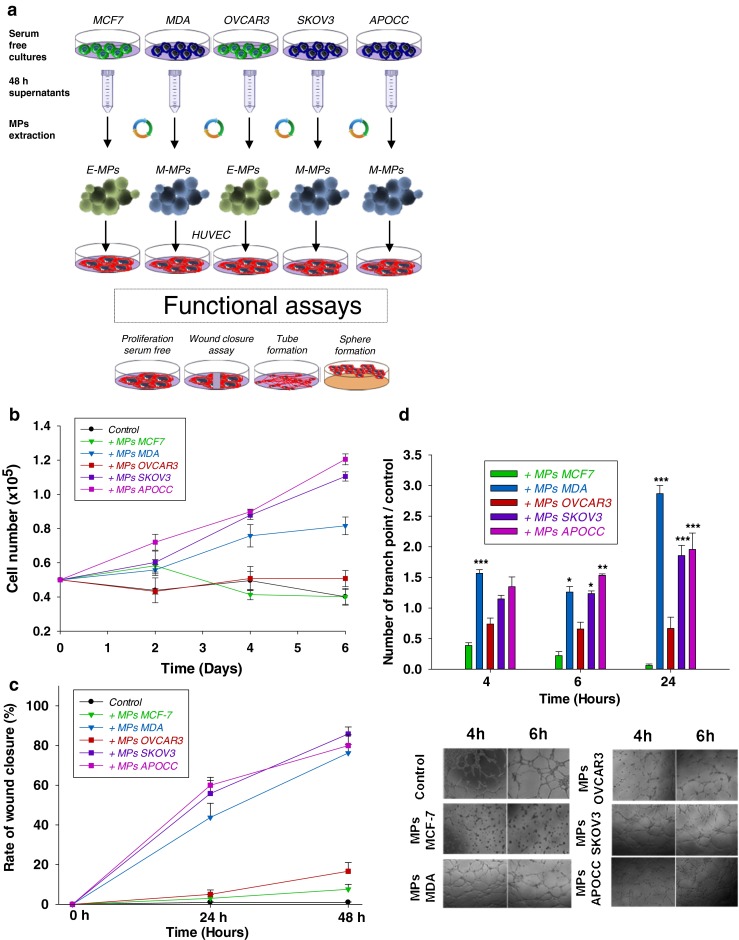
Cancer cells microparticles induce vascular activation. **a** Schematic representation of the experimental design. **b** HUVECs were plated and counted every 2 days in presence or not of MPs from CCs. Only MPs from MDA and Skov3 were able to sustain proliferation of HUVECs. **c** Wound closure assay was performed for HUVECs in presence or absence of CCs-MPs. Only MPs from MDA and Skov3 enhanced HUVECs motility. **d** HUVECs were plated on matrigel layer in presence or not of CC-MPs. Tube formation were quantified at different time. Only MPs from MDA and Skov3 were able to improve the number of tube and their viability. The bottom panel grave representative picture of the tubes at 4 and 6 h after plating. Scale bar: 500 μm

To investigate the functional effect of MPs on endothelial cells, HUVECs were incubated with MPs from MCF7 and OVCAR3, referred as E-MPs (Epithelial-MPs), or MPs from MDA, SKOV3 and APOCC, referred as M-MPs (Mesenchymal-MPs), Fig. 2a.

M-MPs induced increased endothelial proliferation (2.03, 2.75 and 2.91 fold compared to control for MDA, SKOV3 and APOCC, respectively) while E-MPs failed to sustain ECs proliferation (Fig. 2c). M-MPs were also able to stimulate HUVECs motility in serum free condition (76.2, 85.3 and 80.6 fold for MDA, SKOV3 and APOCC, respectively) compared to control and E-MPs (Fig. 2d). Tube formation was observed as early as 4 h after treatment with CCs-MPs; however (i) the number of tubes and the kinetic of tube formation were lower with the E-MPs and (ii) the persistence of tubes was only observed after M-MPs treatments (Fig. 2e). Finally, we illustrated recently the role of the endothelium in lung regeneration within structure called angiospheres where the ECs provided specific angiocrine cues to lung cells [26]. We demonstrated increased angiospheres formation with M-MPs compared to E-MPs (2.5 fold for MDA vs. MCF7 and 2.3 fold for SKOV3 vs. OVCAR3) (Supplementary figure 3).

**Fig. 3:**
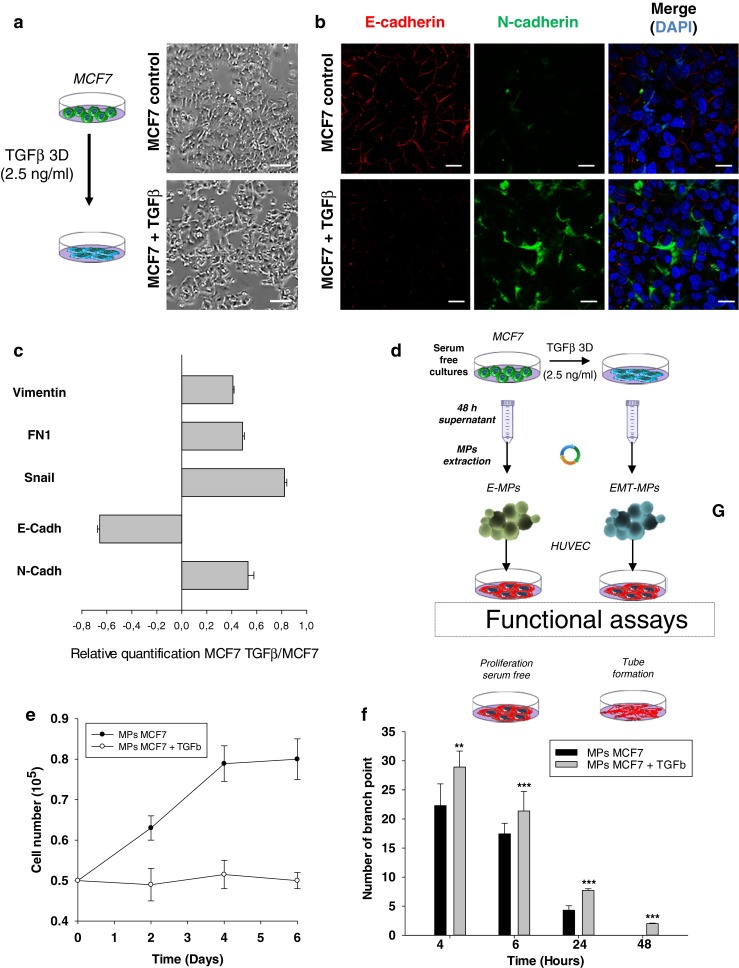
EMT in cancer cells induce a modification in the microparticles released. **a** MCF7 were treated with TGFβ (2.5 ng/ml) during 3 days. Phase contrast microscopy showing significant morphological change concordant with a mesenchymal phenotype. Scale bar 500 μm. **b** After treatment with TFGβ, MCF7 were stained for E-cadherin or N-cadherin and analyzed by confocal microscopy. An increase of N-cadherin staining and a decrease of E-cadherin staining can be observed in the MCF7 treated with TGFβ in comparison to controls. Scale bar: 10 μm. **c** The relative quantification of EMT genes were performed by real-time qPCR on MCF7 after treatment with TGFβ. The mesenchymal genes (N-cadherin, Snail, Fibronectin and Vimentin) were increased compared to the control. E-cadherin expression was decreased. Relative transcript levels are represented as the log_10_ of ratios between the 2 subpopulations of their 2^–ΔΔCp^ real-time PCR values. **d** Schematic representation of the experimental design to assess the functional role of iM-MPs. **e** HUVECs were plated and counted every 2 days in presence of MCF7-MPs or iM-MPs. Only iM-MPs were able to sustain proliferation of HUVECs. **f** HUVECs were plated on matrigel layer in presence of MCF7-MPs or iM-MPs. Only iM-MPs were able to improve the number of tube and their viability

Our data suggested that M-MPs displayed a specific ability to activate ECs compare to E-MPs. However, this could be cell-specific rather than phenotype-related. Therefore we used an in vitro Epithelial to Mesenchymal Transition (EMT) model using TGFβ to investigate the functional modifications of corresponding MPs [35]. MCF7 cells were treated by TGFβ for 3 days until morphological changes appeared (Fig. 3a). EMT was then confirmed by reduced expression of E-cadherin and increased expression of N-cadherin, Vimentin, Fibronectin and the transcription factor SNAIL (Fig. 3b-c). We subsequently starved the EMT cells and isolated the induced mesenchymal MPs (iM-MPs) (Fig. 3d). We showed that the iM-MPs were able to increase both ECs proliferation and branching in a Matrigel assay, compared to the MCF7-MPs (Fig. 3e-f).

### M-MPs induced activation of EC and triggered an Arf6 mediated increase in EC-MPs secretion

**Fig. 4:**
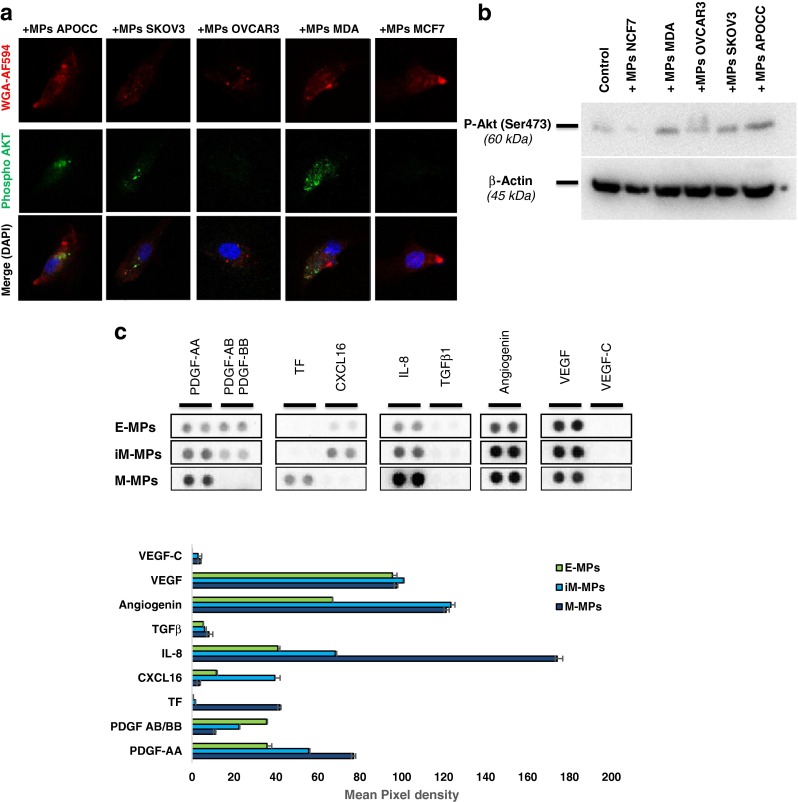
Mensenchymal MPs are able to induce endothelial activation. **a** HUVECs were incubated with MPs from each cancer cells lines during 30 min and Akt activation was analyzed by confocal microscopy and flow cytometry. Prior to confocal microscopy HUVECs were stained with WGA red and nuclei were tagged with DAPI. Only MPs from MDA and SKOV3 were able to induce Akt phosphorylation in HUVECs. Scale bar 20 μm. **b** Western blot for Phospho-Akt (Ser473) confirmed that only MPs from MDA, SKOV3 and APOCC are able to induce phosphorylation of Akt in HUVECS. C. Human angiogenesis array of MPs contents from an E-MPs (MCF7), iM-MPs (MCF7 treated with TGFβ) and M-MPs (MDA) model. The significant protein modification were cut from the full membrane (Supplementary Figure 4) and represent in the top panel. The bottom panel represents the relative quantification of the dot pixel density

Akt phosphorylation is known to be involved in EC proliferation, migration and tube formation [36]. Our findings and a recent study by Kawamoto et al. propose that M-MPs could activate Akt phosphorylation in HUVECs [16]. In concordance with the functional experiments, confocal microscopy and western blot confirmed that only M-MPs and iM-MPs induced Akt phosphorylation in HUVECs (Fig. 4a-b and Supplementary figure 4).

We then investigated the differential expression of pro-angiogenic molecules using a human angiogenesis array on E-MPs (MCF7), iM-MPs (MCF7 treated with TGFβ) and M-MPs (MDA; Fig. 4c for a selected panel and Supplementary figure 5). Interestingly VEFG level was similar in E-MPs, iM-MPs and M-MPs and TGFβ could not be detected. Several major angiogenic molecules displayed differential expression between the different MPs tested. Among them PDGFA, Angiogenin and IL8 were upregulated in both iM-MPs and M-MPs compare to E-MPs. Others such as CXCL16 and TF (Tissue Factor) demonstrated differential expression between iM-MPs and M-MPs. As previously published, TF was present in the M-MPs but we could not detect TF in the E-MPs or iM-MPs [37].

**Fig. 5:**
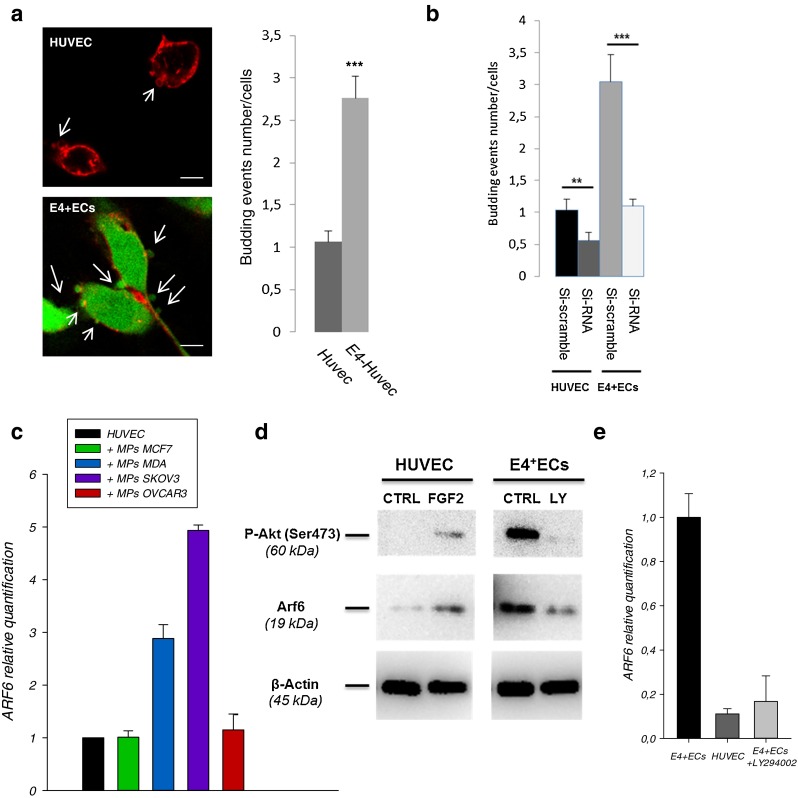
Endothelial activation induces an Arf6 mediated increase in MPs secretion. **a** Budding of MPs was counted in HUVEC and E4+ECs treated or not with M-MPs. Before imaging by confocal microscopy, cells were stained with Alexa Fluor 594 conjugated-wheat germ agglutinin (WGA). 10 areas of the slides were captured and the number of budding by number of cells was quantified. The budding is more important in E4+ECs than HUVECs and is increased in presence of M-MPs. Scale bar: 10 μm. **b** SiRNA of Arf6 decreased the budding in endothelial cells. **c** Arf6 relative expression was evaluated by real-time qPCR. Arf6 expression is increased in HUVECs in presence of M-MPs. **d** Western blot analysis showed that Arf6 expression is dependent of Akt phosphorylation. **f** Arf6 relative expression was evaluated by real-time qPCR. Arf6 expression is more important in E4+ECs than HUVECs and decreased when the cells were treated with an Akt inhibitor (LY294002)

In order to understand the effect of Akt phosphorylation on endothelial cells microparticles machinery we used a model of endothelial cells with autonomous Akt-activation surviving in the absence of FBS and cytokines (HUVECs-E4ORF1, referred to as E4+ECs, Supplementary figure 6) [38]. Live cell imaging showed increased budding of MPs at the membrane of E4+ECs compared to the HUVECs (Fig. 5a). Several genes have been implicated in MPs release such as Rab27, Rab11, Arf6, P53, TSAP6 and DGKA [7,20,39–41]. We performed transcriptomic analysis comparing HUVEC and E4+ECs. There was a moderate increase in expression of Rab27 and Rab11 (1.2 to 1.5 fold respectively). P53, TSAP6 and DGKA expression were not modified. Arf6 expression was increased by 2.1 fold; therefore we investigated the role of Arf6 in our experimental conditions. Arf6 is a GTPase of the Ras superfamily playing a major role in membrane trafficking and MPs secretion [20]. Si-RNA mediated inhibition of Arf6 (Supplementary figure 7 for inhibition efficiency) dramatically reduced endothelial and cancer cells MPs secretion (data not shown). Live cell imaging concordantly showed decreased budding at the membrane of both HUVEC^Arf6-^ and E4+EC^Arf6-^ cells (Fig. 5b). We then showed that Arf6 expression was increased in HUVECs treated with M-MPs compared to E-MPs (Fig. 5c). Finally, as M-MPs induced Akt activation in HUVEC, we investigated the role of Akt activation in Arf6 expression. First we showed that FGF2-mediated EC activation induced concomitant Akt activation and Arf6 expression (Fig. 5d). Concordantly, inhibition of P-Akt by LY294002, in E4+ECs resulted in a decreased expression of Arf6 (Fig. 5d-e).

**Fig. 6:**
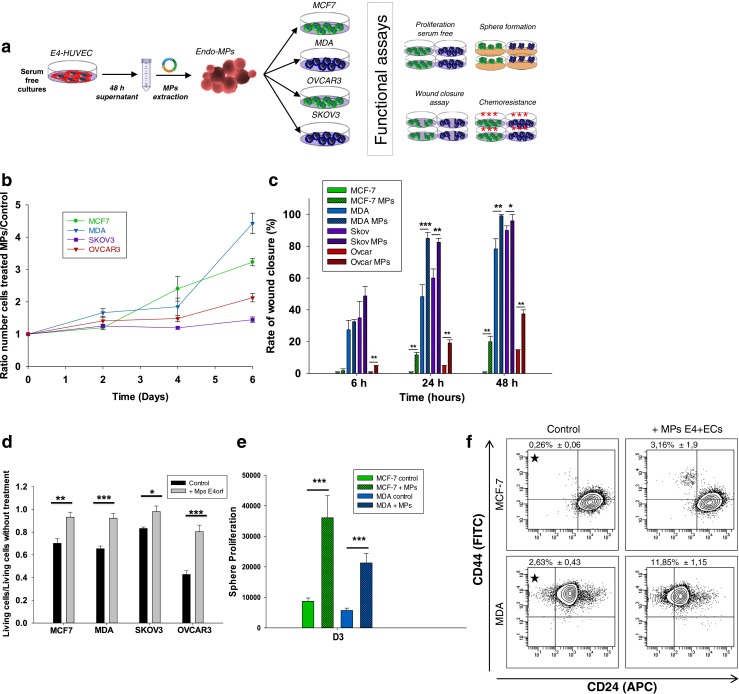
MPs from activated endothelial cells support tumor pro-metastatic phenotype. **a** Schematic representation of the experimental design. **b** CCs were plated and counted every 2 days in presence or not of MPs from E4+ECs during 6 days. EC-MPs were able to sustain proliferation of CCs. **c** Wound closure assay showed that ECs-MPs improved the motility of CCs. **d** Breast CCs were treated with Doxorubicine (10 μM) and ovarian CCs with taxol (10 μM) in presence or absence of EC-MPs. EC-MPs induced chemoresistance of CCs to chemotherapy treatment. **e** Spheroids of CCs were grown in 3D media during 6 days, ECs-MPs sustained the proliferation of CCs spheres. **f** CCs were grown with or without MPs during 4 days. Prior to cytometry analysis, breast CCs were immunostained with CD44 and CD24. Gate of interest are represented with a star on the graph. EC-MPs increase the number of putative cancer stem cells in all CCs population

This suggests that the modulation of endothelial plasticity by CCs may be associated with a modulation of the MPs machinery and the constitution of an endothelial pro-tumoral niche. Therefore we investigated the functional effects of EC-MPs extracted from E4+ECs on cancer cells lines focusing on the following pro-metastatic properties: migration, proliferation, chemoresistance and stemness (Fig. 6a).

### Endothelial cells derived MPs induce a pro-metastatic phenotype

We evaluated cancer cells proliferation in a serum and cytokine-free media where survival and proliferation could only rely on EC-MPs. All treated cell lines demonstrated increased proliferation ranging from 1.45 to 4.5 fold at day 6 (Fig. 6b). They also displayed increased migration ranging from 1.26 to 20 fold (Fig. 6c). We next investigated survival of breast cancer cell lines treated by doxorubicin and ovarian cancer cell lines treated by taxol. The pre-treatment of CCs by EC-MPs induced chemoresistance in all cell lines ranging from 1.2 to 1.95 fold (Fig. 6d).

**Fig. 7:**
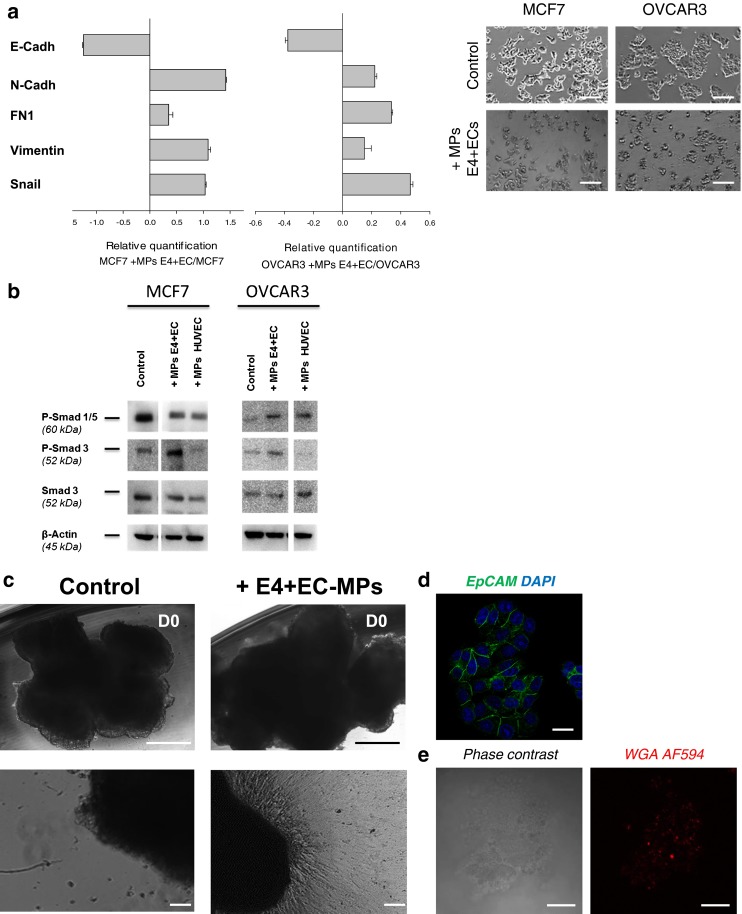
MPs from activated endothelial cells induce EMT in epithelial cancer cells and trigger expansion of cells from ovarian cancer metastatic nodules explants. **a** The relative quantification of EMT genes were performed by real-time qPCR on MCF7 and OVCAR3 after treatment with E4+ECs MPs. The mesenchymal genes (N-cadherin, Snail, Fibronectin and Vimentin) were increased compared to the control. E-cadherin expression was decreased. Relative transcript levels are represented as the log_10_ of ratios between the 2 subpopulations of their 2^–ΔΔCp^ real-time PCR values. Phase contrast microscopy showing significant morphological change concordant with a mesenchymal phenotype (bottom panel). Scale bar 100 μm. **b** Western blot analysis of P-Smad 1/5, P-Smad 3 and Smad3 revealed an implication of P-Smad 3 in the EMT process induced by E4+ECs MPs in MCF7 and OVCAR3 cells. **c** Phase contrast imaging of ovarian cancer explant. Explants of metastatic ovarian cancer nodules were cultured in Petri dishes (top panel). Cells spreading from 3D explants after 7 days of cultured without (bottom left panel) or with E4-ECs MPs stained with AF594-WGA (bottom right panel). Top scale bar: 500 μm. Bottom scale bar: 100 μm. **d** Epcam^+^ outgrowths are observed in the EC-MPs treated group. Scale bar: 25 μm. **e** After 7 days of culture, explants treated with AF594-WGA MPs were collected and analyzed by confocal microscopy. AF594 staining in the explant demonstrated that the MPs have been uptake. Scale bar: 50 μm

Recently the role of cancer propagating cells has been illustrated in both breast and ovarian malignancies [42]. To investigate the effect of EC-MPs on the induction of a propagating phenotype, we used sphere formation assay and flow cytometry. EC-MPs treatment increased CCs sphere formation in all cell lines for both number and size, from 1.3 to 4.13 fold in a serum free 3D media (Fig. 6e and Supplementary figure 7A). To characterize tumor-propagating population by flow cytometry, we used previously described cell surface markers (CD44^+^CD24^low^ for breast cancers, and CD44^+^CD117^+^ for ovarian cancers) [43,44]. Treatment of CCs with EC-MPs increased the putative stem cell population in both breast and ovarian cancer models by 12, 4.5, 2.29 and 1.71 fold for MCF7, MDA, SKOV3 and OVCAR3 respectively (Fig. 7f and Supplementary figure 7B). Interestingly when we performed similar experiments with HUVEC derived MPs normalized on protein quantity and could not demonstrate any significant increase in pro-metastatic phenotype (data not shown).

In order to investigate the occurrence of an EMT, we focused on the two epithelial-like cancer cell lines MCF7 and OVCAR3. We observed an increased expression of mesenchymal markers (N-Cadherin, Vimentin, fibonectin) and transcription factor (Snail) in MCF7 and OVCAR3 after treatment with EC-MPs concordant with the acquisition of a mesenchymal morphology (Fig. 7a). We then investigated the implication of SMADs in OVCAR3 and MCF7 mesenchymal transition (Fig. 7b). MPs from E4+ECs and HUVECs had minor effect on SMAD1 and SMAD5 phosphorylation. However MPs from E4+ECs induced SMAD3 phosphorylation in OVCAR3 cell line.

Finally, we used explants from ovarian cancer metastatic nodules to test the effect of E4+ECs MPs in a 3D tumoral context (Fig. 7c). Ovarian cancer nodules were minced and cultured with or without EC-MPs. After 7 days, we observed more EpCAM^+^ tumor cells outgrowing of the explant in the treated group (Fig. 7d). The confocal microscopy analysis of the explants after 7 days showed cellular uptake of AF594-WGA labeled EC-MPs (Fig. 7e).

## Discussion

In this study, we showed that beyond single factor mediated cross-talk, MPs participate to a complex dialogue between cancer and endothelial cells.

Using endothelial Akt activation as a readout, we were able to differentiate MPs from cells with mesenchymal from cells with epithelial traits. While EC Akt activation by CC-MPs has already been described in the literature [16,34,45–47], this study is the first to address the impact of cancer cells phenotype. The heterogeneity and specific functionality of MPs have been well illustrated in the literature at the protein, mRNA and micro-RNA levels in different cell type in vitro [48–50] and in vivo [51–53]. More recently, few studies highlighted the difference of the MPs extracted from mesenchymal-like cells compared to their epithelial counterparts [37,54,55]. At this stage, the heterogeneity of functional effects, such as endothelial activation, mandates for screening approach where mechanisms of endothelial activation need to be defined.

MPs have been described as shuttle for different types of molecules; we focused here on angiogenic molecules and were able to point out cell type specific MP content as well as shift in content concomitant to phenotype modulation.

The wide range of angiogenic molecules encapsulated in MPs also argues for the synergistic role of multiple mediators. Among the potential candidates, we ruled out the role of VEGF, which had been already detected in MPs from ovarian cancer cell lines [56]. MPs according to their origin demonstrated specific angiogenic profile. TF was only detected in M-MPs concordant with previous study describing its role in MP mediated transfer to endothelial cells [37]. The other angiogenic molecules significantly up regulated in M-MPs and iM-MPs were IL8, angiogenin and PDGF. Addressing their specific role in endothelial cells activation is beyond the scope of our study. Nevertheless, their impact on angiogenesis has been already illustrated. Recently, Martin et al. demonstrated that IL-8 stimulates vascular endothelial growth factor expression and the autocrine activation of VEGFR2 in endothelial cells [57]. Interleukin-8 level are elevated in ascites of patients with ovarian cancer [58]. Its secretion by ovarian cancer cells is also known to increase anchorage-independent growth, proliferation, angiogenic potential, adhesion and invasion [59]. PDGF-AA-induced signaling is through the Ras-MAPK, PI3K/AKT and PLCγ pathways [60]. PDGF role in angiogenesis has been described in diverse context. In surgical flaps, PDGF stimulates angiogenesis in ischemic conditions [61]. In neoplastic context, PDGF has a synergistic role with other angiogenic factors such as FGF or VEGF [62]. Angiogenin is potent angiogenic factor implicated promoting neo-neovascularization [63]. In malignant disease, abnormally high angiogenin levels are observed and might be associated to poor prognosis [64]. Nuclear angiogenin in endothelial cells is necessary for angiogenesis induced by other angiogenic factors including VEGF [65]. In breast cancer, increased angiogenin expression promotes the transition from normal to invasive breast carcinoma [66]. The stroma is now perceived as a complex environment however most studies still focus on single agent effects. This might explain why despite convincing pre-clinical studies, the transition into the clinical setting is to date below expectations. Our data illustrate such complexity, MPs content can concomitantly activate multiple signaling pathways within endothelial cells (data not shown) and blocking a single agent may not efficiently prevent endothelial activation.

Endothelial activation and the role of endothelial paracrine mediators have already been described in angiogenesis [67,68]. Our illustration of arf6 mediated increased budding and MPs excretion once again illustrate the complexity of the cross-talk between tumor and stroma. MPs might play a different role in modifying more broadly the stromal contexture. Arf6 is involved in the shedding of tumors cells derived MPs [20]. Here, we have demonstrated an increased expression of Arf6 during endothelial activation by M-MPs. We showed that Arf6 overexpression in activated ECs is associated with quantitative changes of EC-MPs.

Interestingly, while the activated endothelium MPs were pro-tumoral, regular endothelium MPs did not display the same functionality suggesting an important role of endothelial plasticity. The increased secretion of MPs by cancer cells in the context of cytotoxic stress as well as MPs role in chemoresistance have been illustrated previously [3,69–72]. Our findings of increased chemoresistance and stem cells phenotype in cancer cells population might be of clinical relevance. Once activated, the EC-MPs might allow the occurrence of a chemoresistant stem like cancer cell population through the induction of tumor plasticity. Such resistant clones might play a role in recurrence of disease after treatment.

The role of stromal MPs has not really been clearly described. However, our data suggest an important role of activated EC-MPs in intercellular communication requiring further validation in appropriate in vivo models. Our demonstration of the occurrence of a SMAD3 dependent EMT in the epithelial cancer cell lines advocates for a role of EC-MPs in tumor plasticity. One consequence of such interaction is the occurrence of a self-feeding loop where activated endothelial cells increase mesenchymal phenotype, which in return is able to maintain high activation of endothelial cells.

Concordantly, Peinado et al. recently demonstrated a specific signature of the MPs derived from highly metastatic melanoma cells [6]. They showed the ability of highly metastatic melanoma exosomes to educate bone marrow progenitor cells through MET receptor and therefore to sustain and increase the metastatic behavior of primary tumors to constitute a metastatic niche. This study was the first to demonstrate that transfer of the MET oncoprotein from tumor-derived exosomes to bone marrow progenitor cells promote the metastatic process in vivo.

MPs secretion might be of clinical relevance in other solid tumors. Larger amounts of MPs were observed in ascites from advanced-stage ovarian carcinomas [73]. In vitro, these microparticles stimulated cancer cells migration. In breast cancer, MPs have been isolated in peripheral blood and increased MPs were associated to patients with advanced breast cancer [74]. Moreover, the authors showed that plasma fractions enriched in MPs presented an increased amount of focal adhesion kinase (FAK) and epidermal growth factor receptor (EGFR) foretelling different contents of MPs between the different stages of the tumor.

**Fig. 8:**
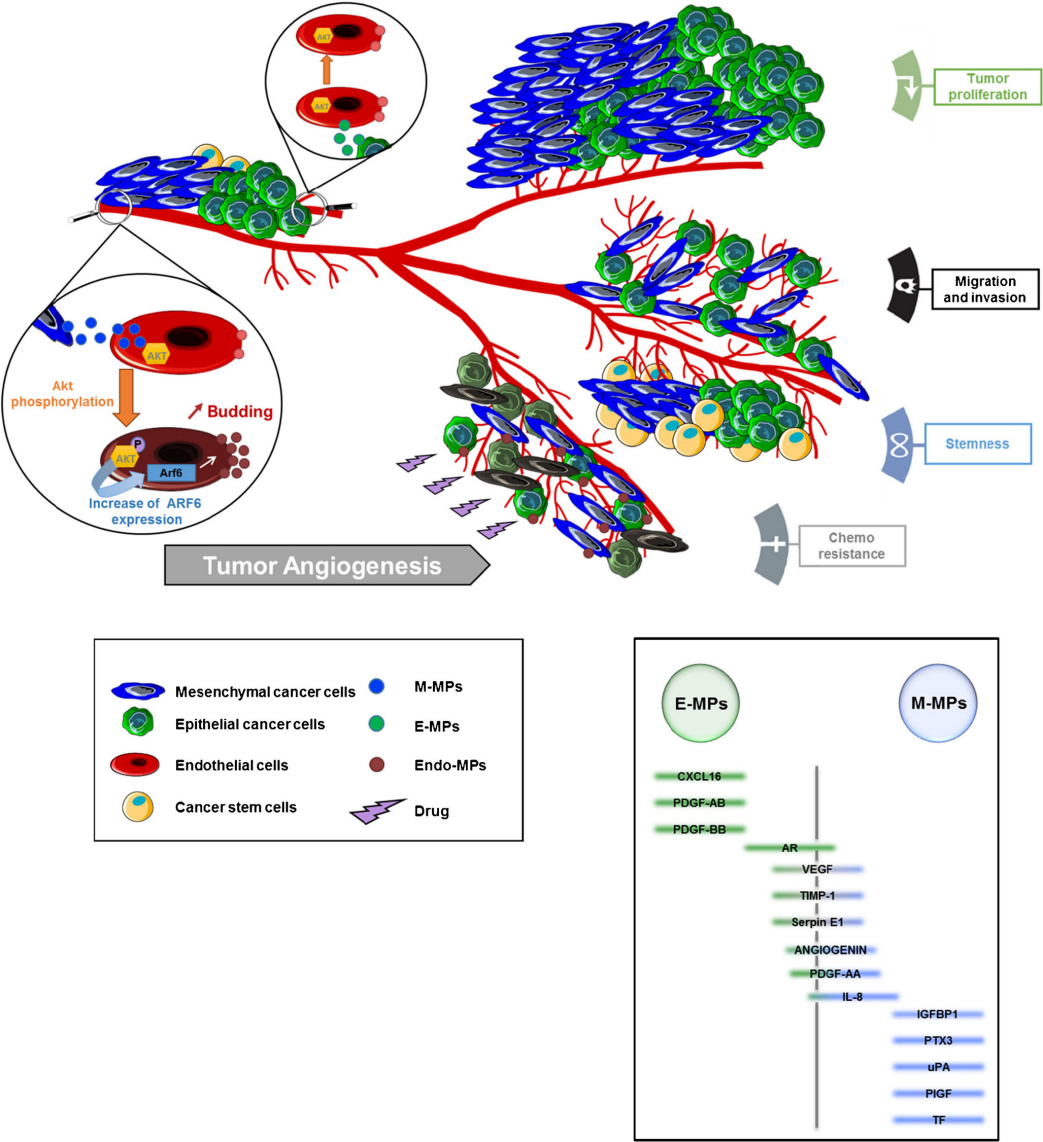
M-MPs activate endothelial cells and functionalize and ARf6 mediated microparticle dependent pro-tumoral niche

Altogether our data propose an active MP mediated cross-talk between endothelial and cancer cells resulting in the constitution of a vascular niche promoting cancer metastatic phenotype (Fig. 8). Our study illustrates the complexity of the interaction between cancer and endothelial cells. Such interactions if confirmed in relevant in vivo models and clinical setting could have potential predictive and therapeutic applications.

## Material and Methods

### Cell Cultures

Ovarian cancer cells lines SKOV3, OVCAR3, breast cancer cell lines MDA-MB231 and MCF7 and Human umbilical cord vein endothelial cells (HUVECs) were purchased from ATCC and cultured following ATCC recommendations (ATCC, Manassas, VA, USA). For 3D cultures, cancer cells were cultivated in ultralow attachment plate (Corning) in DMEM/F12 (1:1) (Hyclone) basal media supplemented with 2 mM L-Glutamine (Hyclone), 1x Non-Essential Amino Acid (NEAA) (Hyclone), PenStrepAmpB (Sigma), 20 ng/ml basic Fibroblast Growth Factor (bFGF) (Peprotech), 20 ng/ml Epidermal Growth Factor (EGF) (Peprotech), 5 μg/ml Insulin (Sigma), 2 % B27 supplements (Invitrogen) and 4 % basement matrigel (BD Biosciences). Cultures were incubated in humidified 5 % CO2 incubators at 37 °C and the media was replaced every 3 days. E4orf1 transfected HUVEC (E4+EC) were obtained as previously described [38]. Cells were cultured in endothelial cell growth medium (Medium 199, 20 % (v/v) fetal bovine serum (FBS), 20 μg ml–1 endothelial cell growth supplement (Hallway), 1 % (v/v) antibiotics (Hallway), and 20 units ml–1 heparin). Cells were serum starved 24 h before isolation of micro-particles. In the E4+EC model the transfection of the adenoviral cassette E4orf1 in HUVECs provides low level of Akt activation allowing the use of serum-free, cytokine-free media without inducing immortalization nor altering the endothelial phenotype [38].

### Microparticles purification

MPs were isolated as previously described [3,75]. Briefly 48-h-supernatants of 80 % serum-starved confluent tumor or endothelial cells were collected, and sequentially centrifuged (4 °C) at 300 g for 10 min, 800 g for 10 min, and then at 3000 g for 15 min. MPs were then pelleted at 100,000 g for 1 h, and washed once in PBS and centrifuged again at 100,000 g for 1 h. The final pellet containing purified MPs was either re-suspended in media for treatment of cell cultures or lysed for protein extraction or labeled for cytometry analysis or microscopy imaging. The protein concentrations of MPs were measured by Bradford assay (Biorad). Cancer cells MPs will be referred to as CC-MPs and endothelial cells MPs as (EC-MPs)

### Migration assay

Migration was assessed by wound closure assay as previously described [76]. Cells cultured at confluence in 24-well plates were scratched with a small tip along the ruler. Cells were then cultured for 6, 24 or 48 h in starvation media with or without MPs. The distances between the edges of the scratch were measured at 0 h and 6, 24 or 48 h after scratching. Data are represented as rate of wound closure.

### Sphere formation assay

Breast and ovarian cancer cell lines MDA-MB231, MCF-7, SKOV3, and OVCAR3 were dissociated into single cell suspension by trypsinization and further sieving through 40-um cell strainers. They were then suspended in 3D media containing DMEM F-12 supplemented with 2 % B27, 20 ng/mL EGF, 20 ng/mL bFGF, and 5 μg/mL insulin. 5000 cells/well were cultured in ultralow attachment plates and were grown with and without EC-MPs in a humidified incubator at 37 °C and 5 % CO2. Mixed spheroids were formed by simultaneous co-culture of cancer cells and E4+ECs. Primary spheres started to form at day 3 and maintained up to days 7 with addition of new MPs to treated spheres every 2 days concomitantly to the change of media. To make secondary spheres, primary spheres were dissociated into single cell suspension and plated at 5000 cells/well as mentioned above. The rate of sphere proliferation with and without microparticles was analyzed by ImageJ64 software based on the area covered by spheres in each well.

### Tube formation assay

A Matrigel-based capillary-genesis assay was performed on E4+EC to assess the ability of these cells to form an organized tubular network as previously described [77]. E4+EC were starved in M199 for 6 h then 100,000 cells were cultured on 250 μl of Matrigel (BD bioscience) in tube assay medium (Medium 199, 0.2 % (v/v) fetal calf serum (FCS), 10 ng ml–1 FGF2 and 20 U ml − 1 heparin). The degree of tube formation was quantified at different time-points by measuring the intersection of tubes in three randomly chosen fields from each well using Image J.

### Flow cytometry

Fluorescence (FL) was quantified on a SORP FACSAria2 (BD Biosciences). Data were processed with FACSDiva 6.3 software (BD Biosciences). Doublets were excluded by FSC-W x FSC-H and SSC-W x SSC-H analysis; eGFP fluorescence, CD44 FITC conjugated (BD Biosciences) and phospho AKT FITC conjugated (S473, cells signaling) were acquired with 488 nm blue laser and 510/50 nm emission, CD24 APC conjugated (BD Biosciences) was acquired with 647 nm red laser and 670/14 nm emission. Charts display the median of fluorescence intensity (mfi) relative to control. Single stained channels were used for compensation and fluorophore minus one (FMO) controls were used for gating. 20,000 events were acquired per sample.

### Quantification of MP uptake

To quantify MP uptake we used stained MPs (WGA AF594) and treated cell cultures for 6 or 24 h at 37 °C or 4 °C. Then cells were harvested and single cell suspension was analyzed by flow cytometry or fixed for confocal microscopy imaging.

### Confocal Microscopy

Tumor and endothelial cells treated with MPs as detailed in specific experiments were fixed in 3.7 % formaldehyde. Slides were mounted in a mounting media SlowFade® Gold Antifade Reagent with DAPI (Invitrogen). Live-cell microscopy was used to analyze co-culture of endothelial and tumor cells. Cells were labeled with 1 mg/ml Alexa Fluor® 594 conjugated wheat germ agglutinin (WGA) (Invitrogen) at 5 μg/ml for 10 min at 37 °C in the dark. Fluorescence Imaging was performed using a Zeiss confocal Laser Scanning Microscope 710 (Carl Zeiss). Post-acquisition image analysis was performed with Zeiss LSM Image Browser Version 4.2.0.121 (Carl Zeiss).

### Western Blot analysis

Cells were lysed in 20 mM Tris–HCl buffer (pH 8.0) containing 100 mM NaCl, 2 mM EDTA, 1 % NP40, 50 mM β-glycerophosphate, 1 mM NaV0_4_, 40 mM Na I, 1 mM DTT and protease inhibitor. Protein lysates were placed on ice for 30 min, vortexed every 5 min, and cleared by centrifugation at 3,500 g for 15 min at 4 °C. The supernatants were collected and frozen at -80 °C until analysis. Protein concentration in extracts was determined using the Bradford method. Aliquots (40 μg) of total proteins extracted from cultured cells were subjected to electrophoresis through a 10 % SDS–polyacrylamide gel at a constant voltage of 70 V for 30 min, then 140 V for 75 min in running buffer (25 mM Tris–HCl pH 8.3, 192 mM glycine, and 0.1 % SDS).

Proteins were then transferred to nitrocellulose membrane at 70 V in transfer buffer (25 mM Tris–HCl pH 8.3, 150 mM glycine and 5 % v/v methanol) for 90 min. The nitrocellulose membrane was blocked with 5 % BSA in Tris-buffered saline (TBS) (20 mM Tris pH 7.6, 137 mM NaCl) for 1 h. Immunostaining was carried out using a goat monoclonal ARF6 and PhosphoAKT antibody (1/1000, Cells signalling) and a secondary polyclonal mouse anti-goat antibody HRP conjugated (1/2000, cell signalling). Blots were developed using HRP and chemiluminescent peroxidase substrate (#CPS1120, Sigma). Data were collected using Geliance CCD camera (Perkin Elmer), and analyzed using ImageJ software (NIH).

### RT-PCR analysis

Total RNA was extracted from cells cultures using Trizol. After genomic DNA removal by DNase digestion (Turbo DNA free kit, Applied Biosystems), total RNA (1 μg) was reverse transcribed with oligodT (Promega) using the Superscript III First-Strand Synthesis SuperMix (Invitrogen). PCR analysis was performed with a MasterCycler apparatus (Eppendorf) from 2 μL of cDNA using RT^2^ qPCR Primer Assay for Human ARF6 (Qiagen, PPH10416A) and primers from IDT for the others genes (Table 1).

**Table 1:** Primers sequence used for RT-PCR

Primer name	Forward	Reverse
FN1	CAGTGGGAGACCTCGAGAAG	TCCCTCGGAACATCAGAAAC
Snail	CCTCCCTGTCAGATGAGGAC	CCAGGCTGAGGTATTCCTTG
N-Cad	CCGAGATGGGGTTGATAATG	ACAGTGGCCACCTACAAAGG
E-Cad	TGCCCAGAAAATGAAAAG	GTGTATGTGGCAATGCGTTC
Vim	GAGAACTTTGCCGTTGAAGC	GCTTCCTGTAGGTGGCAATC
GAPDH	AGCCACATCGCTCAGACAC	GCCCAATACGACCAAATCC

After an initial denaturation step at 94 °C for 2 min, 35 cycles were performed including a denaturation step at 94 °C for 30 s, annealing at 55 °C for 30 s and extension at 72 °C for 30 s. The final extension step was continued for 5 min. GAPDH amplification was used as a qualitative control. DNA was omitted in non-template control (NTC). The absence of genomic DNA traces in RNA samples was checked by performing amplification from RNA without previous reverse-transcription. PCR products were analysed on a 1 % agarose gel and visualized by SYBR staining (SYBR Safe DNA gel stain, Invitrogen). Amplicon length was evaluated using a standard molecular weight marker (100 bp DNA ladder, Promega).

### SiRNA treatment

siRNA against human Arf6 (Santa Cruz biotechnology) were introduced into cells by lipid mediated transfection using siRNA transfection medium, reagent and duplex (Santa Cruz biotechnology) following manufacturer recommendations. Briefly the day before transfection cells were patted at 2,5 .10^5^ cells per well in 2 ml antibiotic-free normal growth medium supplemented with FBS. Cells were incubated until they reach 60-80 % confluence. The duplex solution containing the siRNA is then added to the cells. After 5 to 7 h, antibiotic are added in each well and the cells are incubated for 24 h more. The media is then replaced by normal growth media and cells are used for experiments and assay by RT-PCR to analyze the expression of ARF6 gene.

### Chemoresistance and cell Viability study (MTT Assay)

Viability of cells was examined with an MTT assay. 24 h after treatment with doxorubicin or taxol, 10 % of MTT reagent was added to each well to a final concentration of 500 μg/ml, and the cells were incubated for 4 h at 37 °C. 100 μl of DMSO were added to each well. Optical density was read at 570 nm versus 630 with an EnVision multilabel reader (PerkinElmer). 3 triplicates were performed per condition.

### Explant culture

Tumor material from patients presenting advanced ovarian carcinoma Stage IIIC were included in this study (IRB Number: #9161/2010, “Isolation and characterization of cancer stem cells”). During debulking surgery metastatic nodules were removed and processed as follows. Upon serial washing with PBS and red blood cell Lysis buffer (eBiosciences, San Diego, USA) nodules were minced and cultured with or without MPs extracted from E4+ECs in DMEM high glucose (Hyclone, Thermo Scientific) supplemented with 20 % FBS (Hyclone, Thermo Scientific), 1 % Penicillin-Streptomycin-Amphotericyn B solution (Sigma), 2 mM L-glutamine (Sigma), 1X Non-Essential Amino-Acid (Hyclone, Thermo Scientific).

### Statistical analysis

All quantitative data were expressed as mean ± standard error of the mean (SEM). Statistical analysis was performed by using SigmaPlot 11 (Systat Software Inc., Chicago, IL). A Shapiro-Wilk normality test, with a *p* = 0.05 rejection value, was used to test normal distribution of data prior further analysis. All pairwise multiple comparisons were performed by one way ANOVA followed by Holm-Sidak posthoc tests for data with normal distribution or by Kruskal-Wallis analysis of variance on ranks followed by Tukey posthoc tests, in case of failed normality test. Paired comparisons were performed by Student’s t-tests or by Mann–Whitney rank sum tests in case of unequal variance or failed normality test. Statistical significance was accepted for *p* < 0.05 (*), *p* < 0.01 (**) or *p* < 0.001 (***). All experiments were performed in triplicates.

## Electronic supplementary material

Below is the link to the electronic supplementary material.

Supplementary Figure 1 Actin co-localization with MPs. eGFP-E4 + ECs were co-cultured with MDA or OVCAR3 cells for 3 days. Before imaging by confocal microscopy, fixed cells were stained with DAPI and AlexaFluor 647 conjugated-phalloidin. Fixed cells were stained with WGA, DAPI and AlexaFluor 647 conjugated-phalloidin. Arrows indicates area where eGFP-E4 + ECs-MPs (green) co-localize with patches of actin (red). Scale bar 10 μm (PDF 64.5 kb)

Supplementary Figure 2 MPs uptake is an active process depending of temperature. **A**. MPs from E4 + ECs were extracted from 80 % confluent cells and labeled with Alexa Fluor 594 conjugated-wheat germ agglutinin (WGA). Cancer cells lines were incubated with E4 + ECs-MPs for 24 h at 37°c or 4°c. CCs were tagged prior to co-culture with the persistent fluorescent probe CellTracker Green. MPs uptake by the cells only occurs at 37°c. At 4°c, MPs aggregate at the plasma membrane. MPs uptake quantification was performed by flow cytometry (bottom panel). Scale bar 20 μm. **B**. MPs from all cancer cells lines were extracted from 80 % confluent cells and labeled with Alexa Fluor 594 conjugated-wheat germ agglutinin (WGA). ECs were incubated with the MPs of each cancer cell lines for 24 h at 37°c or 4°c. Quantification of MPs uptake was done by flow cytometry (bottom panel). Scale bar 20 μm. (PDF 392 kb)

Supplementary Figure 3 MPs of cancer cell lines increase angiosphere formation. Spheroids of HUVECs were grown in 3D media during 6 days with or without CCs-MPs. Only MPs from MDA and Skov3 sustain the proliferation of HUVECs spheres. (PDF 63.6 kb)

Supplementary Figure 4 iM-MPs induce Akt phosphorylation in HUVECs. HUVECs were incubated during 30 min with MPs from MCF7 (MCF7-MPs) or MCF7 treated with TGFβ (iM-MPs) and analyzed by confocal microscopy. Only iM-MPs were able to induce phospho-AKT in HUVECs. Scale bar 20 μm. (PDF 50.4 kb)

Supplementary Figure 5 Whole membrane of the Human Angiogenesis Array. Representation of the membrane used for the representation and quantification of angiogenesis-related proteins presented in the figure 5 C. The coordinates and their target were given accordingly to the provider protocol. (PDF 806 kb)

Supplementary Figure 6 E4 + ECs display an autonomous Akt phosphorylation. Akt phosphorylation level of E4 + ECs in comparison to the HUVECS by confocal microscopy (left panel) and flow cytometry (right panel). (PDF 62.3 kb)

Supplementary Figure 7 Expression of ARF6 after siRNA treatment. The relative quantification of ARF6 gene was performed by RT-PCR on HUVEC and E4ORF after treatment with SiRNA for ARF6 or the siRNA scramble (control). The ARF6 expression is completely inhibited up to 6 days after the treatment with siRNA. (PDF 33.7 kb)

Supplementary Figure 8 Implication of EC-MPs in cancer stemness. **A**. Spheroids of CCs were grown in 3D media during 6 days with or without E4 + ECs-MPs. E4 + ECs-MPs sustain the proliferation of CCs spheres. **B**. CCs were grown with or without E4 + ECs-MPs during 4 days. Before cytometry analysis, ovarian CCs were immunostained with CD44 and CD117. Gate of interest are represented with a star on the graph. E4 + ECs-MPs increase the number of putative cancer stem cells in all CCs population. (PDF 73.4 kb)

## References

[1] Karnoub AE, Dash AB, Vo AP, Sullivan A, Brooks MW, Bell GW, Richardson AL, Polyak K, Tubo R, Weinberg RA (2007). Mesenchymal stem cells within tumour stroma promote breast cancer metastasis. Nature.

[2] Lis R, Touboul C, Mirshahi P, Ali F, Mathew S, Nolan DJ, Maleki M, Abdalla SA, Raynaud CM, Querleu D, Al-Azwani E, Malek J, Mirshahi M, Rafii A (2011). Tumor associated mesenchymal stem cells protects ovarian cancer cells from hyperthermia through CXCL12. Int J Cancer.

[3] Pasquier J, Galas L, Boulange-Lecomte C, Rioult D, Bultelle F, Magal P, Webb G, Le Foll F (2012). Different modalities of intercellular membrane exchanges mediate cell-to-cell p-glycoprotein transfers in MCF-7 breast cancer cells. J Biol Chem.

[4] Pasquier J, Magal P, Boulange-Lecomte C, Webb G, Le Foll F (2011). Consequences of cell-to-cell P-glycoprotein transfer on acquired multidrug resistance in breast cancer: a cell population dynamics model. Biol Direct.

[5] Rafii A, Mirshahi P, Poupot M, Faussat AM, Simon A, Ducros E, Mery E, Couderc B, Lis R, Capdet J, Bergalet J, Querleu D, Dagonnet F, Fournie JJ, Marie JP, Pujade-Lauraine E, Favre G, Soria J, Mirshahi M (2008). Oncologic trogocytosis of an original stromal cells induces chemoresistance of ovarian tumours. PLoS One.

[6] Peinado H, Aleckovic M, Lavotshkin S, Matei I, Costa-Silva B, Moreno-Bueno G, Hergueta-Redondo M, Williams C, Garcia-Santos G, Ghajar C, Nitadori-Hoshino A, Hoffman C, Badal K, Garcia BA, Callahan MK, Yuan J, Martins VR, Skog J, Kaplan RN, Brady MS, Wolchok JD, Chapman PB, Kang Y, Bromberg J, Lyden D (2012). Melanoma exosomes educate bone marrow progenitor cells toward a pro-metastatic phenotype through MET. Nat Med.

[7] Thery C, Ostrowski M, Segura E (2009). Membrane vesicles as conveyors of immune responses. Nat Rev Immunol.

[8] Thery C, Zitvogel L, Amigorena S (2002). Exosomes: composition, biogenesis and function. Nat Rev Immunol.

[9] Pan BT, Johnstone RM (1983). Fate of the transferrin receptor during maturation of sheep reticulocytes in vitro: selective externalization of the receptor. Cell.

[10] D’Souza-Schorey C, Clancy JW (2012). Tumor-derived microvesicles: shedding light on novel microenvironment modulators and prospective cancer biomarkers. Genes Dev.

[11] Eldh M, Ekstrom K, Valadi H, Sjostrand M, Olsson B, Jernas M, Lotvall J (2010). Exosomes communicate protective messages during oxidative stress; possible role of exosomal shuttle RNA. PLoS One.

[12] Pelchen-Matthews A, Raposo G, Marsh M (2004). Endosomes, exosomes and Trojan viruses. Trends Microbiol.

[13] Wolfers J, Lozier A, Raposo G, Regnault A, Thery C, Masurier C, Flament C, Pouzieux S, Faure F, Tursz T, Angevin E, Amigorena S, Zitvogel L (2001). Tumor-derived exosomes are a source of shared tumor rejection antigens for CTL cross-priming. Nat Med.

[14] Gong J, Jaiswal R, Mathys JM, Combes V, Grau GE, Bebawy M (2012). Microparticles and their emerging role in cancer multidrug resistance. Cancer Treat Rev.

[15] Bebawy M, Combes V, Lee E, Jaiswal R, Gong J, Bonhoure A, Grau GE (2009). Membrane microparticles mediate transfer of P-glycoprotein to drug sensitive cancer cells. Leukemia.

[16] Kawamoto T, Ohga N, Akiyama K, Hirata N, Kitahara S, Maishi N, Osawa T, Yamamoto K, Kondoh M, Shindoh M, Hida Y, Hida K (2012). Tumor-derived microvesicles induce proangiogenic phenotype in endothelial cells via endocytosis. PLoS One.

[17] Svensson KJ, Kucharzewska P, Christianson HC, Skold S, Lofstedt T, Johansson MC, Morgelin M, Bengzon J, Ruf W, Belting M (2011). Hypoxia triggers a proangiogenic pathway involving cancer cell microvesicles and PAR-2-mediated heparin-binding EGF signaling in endothelial cells. Proc Natl Acad Sci U S A.

[18] Li XB, Zhang ZR, Schluesener HJ, Xu SQ (2006). Role of exosomes in immune regulation. J Cell Mol Med.

[19] Pap E (2011). The role of microvesicles in malignancies. Adv Exp Med Biol.

[20] Muralidharan-Chari V, Clancy JW, Sedgwick A, D’Souza-Schorey C (2010). Microvesicles: mediators of extracellular communication during cancer progression. J Cell Sci.

[21] Folkman J (2006). Angiogenesis. Ann Rev Med.

[22] Jayson GC, Hicklin DJ, Ellis LM (2012). Antiangiogenic therapy–evolving view based on clinical trial results. Nat Rev Clin Oncol.

[23] Rapisarda A, Melillo G (2012). Overcoming disappointing results with antiangiogenic therapy by targeting hypoxia. Nat Rev Clin Oncol.

[24] Ebos JM, Kerbel RS (2011). Antiangiogenic therapy: impact on invasion, disease progression, and metastasis. Nat Rev Clin Oncol.

[25] Sitohy B, Nagy JA, Dvorak HF (2012). Anti-VEGF/VEGFR therapy for cancer: reassessing the target. Cancer Res.

[26] Ding BS, Nolan DJ, Guo P, Babazadeh AO, Cao Z, Rosenwaks Z, Crystal RG, Simons M, Sato TN, Worgall S, Shido K, Rabbany SY, Rafii S (2011). Endothelial-derived angiocrine signals induce and sustain regenerative lung alveolarization. Cell.

[27] Maretzky T, Evers A, Zhou W, Swendeman SL, Wong PM, Rafii S, Reiss K, Blobel CP (2011). Migration of growth factor-stimulated epithelial and endothelial cells depends on EGFR transactivation by ADAM17. Nat Commun.

[28] Pasquier J, Guerrouahen BS, Al Thawadi H, Ghiabi P, Maleki M, Abu-Kaoud N, Jacob A, Mirshahi M, Galas L, Rafii S, Le Foll F, Rafii A (2013). Preferential transfer of mitochondria from endothelial to cancer cells through tunneling nanotubes modulates chemoresistance. J Transl Med.

[29] Pasquier J, Rafii A (2013). Role of the microenvironment in ovarian cancer stem cell maintenance. Biomed Res Int.

[30] Folkman J (2001). Angiogenesis-dependent diseases. Semin Oncol.

[31] Nguyen M, Watanabe H, Budson AE, Richie JP, Hayes DF, Folkman J (1994). Elevated levels of an angiogenic peptide, basic fibroblast growth factor, in the urine of patients with a wide spectrum of cancers. J Nat Cancer Inst.

[32] Rak JW, Hegmann EJ, Lu C, Kerbel RS (1994). Progressive loss of sensitivity to endothelium-derived growth inhibitors expressed by human melanoma cells during disease progression. J Cell Physiol.

[33] Lu J, Ye X, Fan F, Xia L, Bhattacharya R, Bellister S, Tozzi F, Sceusi E, Zhou Y, Tachibana I, Maru DM, Hawke DH, Rak J, Mani SA, Zweidler-McKay P, Ellis LM (2013). Endothelial cells promote the colorectal cancer stem cell phenotype through a soluble form of Jagged-1. Cancer Cell.

[34] Antonyak MA, Li B, Boroughs LK, Johnson JL, Druso JE, Bryant KL, Holowka DA, Cerione RA (2011). Cancer cell-derived microvesicles induce transformation by transferring tissue transglutaminase and fibronectin to recipient cells. Proc Natl Acad Sci U S A.

[35] Katsuno Y, Lamouille S, Derynck R (2013). TGF-beta signaling and epithelial-mesenchymal transition in cancer progression. Curr Opin Oncol.

[36] Weng L, Enomoto A, Ishida-Takagishi M, Asai N, Takahashi M (2010). Girding for migratory cues: roles of the Akt substrate Girdin in cancer progression and angiogenesis. Cancer Sci.

[37] Garnier D, Magnus N, Lee TH, Bentley V, Meehan B, Milsom C, Montermini L, Kislinger T, Rak J (2012). Cancer cells induced to express mesenchymal phenotype release exosome-like extracellular vesicles carrying tissue factor. J Biol Chem.

[38] Seandel M, Butler JM, Kobayashi H, Hooper AT, White IA, Zhang F, Vertes EL, Kobayashi M, Zhang Y, Shmelkov SV, Hackett NR, Rabbany S, Boyer JL, Rafii S (2008). Generation of a functional and durable vascular niche by the adenoviral E4ORF1 gene. Proc Natl Acad Sci U S A.

[39] Muralidharan-Chari V, Clancy J, Plou C, Romao M, Chavrier P, Raposo G, D’Souza-Schorey C (2009). ARF6-regulated shedding of tumor cell-derived plasma membrane microvesicles. Current Biol: CB.

[40] Ostrowski M, Carmo NB, Krumeich S, Fanget I, Raposo G, Savina A, Moita CF, Schauer K, Hume AN, Freitas RP, Goud B, Benaroch P, Hacohen N, Fukuda M, Desnos C, Seabra MC, Darchen F, Amigorena S, Moita LF, Thery C (2010). Rab27a and Rab27b control different steps of the exosome secretion pathway. Nat Cell Biol.

[41] Bianco F, Perrotta C, Novellino L, Francolini M, Riganti L, Menna E, Saglietti L, Schuchman EH, Furlan R, Clementi E, Matteoli M, Verderio C (2009). Acid sphingomyelinase activity triggers microparticle release from glial cells. EMBO J.

[42] Tysnes BB (2010). Tumor-initiating and -propagating cells: cells that we would like to identify and control. Neoplasia.

[43] Al-Hajj M, Wicha MS, Benito-Hernandez A, Morrison SJ, Clarke MF (2003). Prospective identification of tumorigenic breast cancer cells. Proc Natl Acad Sci U S A.

[44] Bapat SA, Mali AM, Koppikar CB, Kurrey NK (2005). Stem and progenitor-like cells contribute to the aggressive behavior of human epithelial ovarian cancer. Cancer Res.

[45] Al-Nedawi K, Meehan B, Kerbel RS, Allison AC, Rak J (2009). Endothelial expression of autocrine VEGF upon the uptake of tumor-derived microvesicles containing oncogenic EGFR. Proc Natl Acad Sci U S A.

[46] Kucharzewska P, Christianson HC, Welch JE, Svensson KJ, Fredlund E, Ringner M, Morgelin M, Bourseau-Guilmain E, Bengzon J, Belting M (2013). Exosomes reflect the hypoxic status of glioma cells and mediate hypoxia-dependent activation of vascular cells during tumor development. Proc Natl Acad Sci U S A.

[47] Corrado C, Flugy AM, Taverna S, Raimondo S, Guggino G, Karmali R, De Leo G, Alessandro R (2012). Carboxyamidotriazole-orotate inhibits the growth of imatinib-resistant chronic myeloid leukaemia cells and modulates exosomes-stimulated angiogenesis. PLoS One.

[48] Park JO, Choi DY, Choi DS, Kim HJ, Kang JW, Jung JH, Lee JH, Kim J, Freeman MR, Lee KY, Gho YS, Kim KP (2013). Identification and characterization of proteins isolated from microvesicles derived from human lung cancer pleural effusions. Proteomics.

[49] Raimondo F, Morosi L, Chinello C, Magni F, Pitto M (2011). Advances in membranous vesicle and exosome proteomics improving biological understanding and biomarker discovery. Proteomics.

[50] Chlebowski A, Lubas M, Jensen TH, Dziembowski A (2013). RNA decay machines: the exosome. Biochim Biophys Acta.

[51] Hogan MC, Johnson KL, Zenka RM, Cristine Charlesworth M, Madden BJ, Mahoney DW, Oberg AL, Huang BQ, Leontovich AA, Nesbitt LL, Bakeberg JL, McCormick DJ, Robert Bergen H, Ward CJ (2013). Subfractionation, characterization, and in-depth proteomic analysis of glomerular membrane vesicles in human urine. Kidney Int.

[52] Inal JM, Kosgodage U, Azam S, Stratton D, Antwi-Baffour S, Lange S (2013). Blood/plasma secretome and microvesicles. Biochim Biophys Acta.

[53] Chaput N, Thery C (2011). Exosomes: immune properties and potential clinical implementations. Sem Immunopathol.

[54] Ji H, Greening DW, Barnes TW, Lim JW, Tauro BJ, Rai A, Xu R, Adda C, Mathivanan S, Zhao W, Xue Y, Xu T, Zhu HJ, Simpson RJ (2013). Proteome profiling of exosomes derived from human primary and metastatic colorectal cancer cells reveal differential expression of key metastatic factors and signal transduction components. Proteomics.

[55] Tauro BJ, Greening DW, Mathias RA, Mathivanan S, Ji H, Simpson RJ (2013). Two distinct populations of exosomes are released from LIM1863 colon carcinoma cell-derived organoids. Mol Cell Proteomics : MCP.

[56] Taraboletti G, D’Ascenzo S, Giusti I, Marchetti D, Borsotti P, Millimaggi D, Giavazzi R, Pavan A, Dolo V (2006). Bioavailability of VEGF in tumor-shed vesicles depends on vesicle burst induced by acidic pH. Neoplasia.

[57] Martin D, Galisteo R, Gutkind JS (2009). CXCL8/IL8 stimulates vascular endothelial growth factor (VEGF) expression and the autocrine activation of VEGFR2 in endothelial cells by activating NFkappaB through the CBM (Carma3/Bcl10/Malt1) complex. J Biol Chem.

[58] Matte I, Lane D, Laplante C, Rancourt C, Piche A (2012). Profiling of cytokines in human epithelial ovarian cancer ascites. Am J Cancer Res.

[59] Wang Y, Xu RC, Zhang XL, Niu XL, Qu Y, Li LZ, Meng XY (2012). Interleukin-8 secretion by ovarian cancer cells increases anchorage-independent growth, proliferation, angiogenic potential, adhesion and invasion. Cytokine+.

[60] Andrae J, Gallini R, Betsholtz C (2008). Role of platelet-derived growth factors in physiology and medicine. Genes Dev.

[61] Machens HG, Morgan JR, Berthiaume F, Stefanovich P, Siemers F, Krapohl B, Berger A, Mailander P (2002). Platelet-derived growth factor-AA-mediated functional angiogenesis in the rat epigastric island flap after genetic modification of fibroblasts is ischemia dependent. Surgery.

[62] Zhang J, Cao R, Zhang Y, Jia T, Cao Y, Wahlberg E (2009). Differential roles of PDGFR-alpha and PDGFR-beta in angiogenesis and vessel stability. FASEB J : Off Pub Fed Am Soc Exp Biol.

[63] Li S, Hu GF (2012). Emerging role of angiogenin in stress response and cell survival under adverse conditions. J Cell Physiol.

[64] Tello-Montoliu A, Patel JV, Lip GY (2006). Angiogenin: a review of the pathophysiology and potential clinical applications. J Thromb Haemos : JTH.

[65] Kishimoto K, Liu S, Tsuji T, Olson KA, Hu GF (2005). Endogenous angiogenin in endothelial cells is a general requirement for cell proliferation and angiogenesis. Oncogene.

[66] Campo L, Turley H, Han C, Pezzella F, Gatter KC, Harris AL, Fox SB (2005). Angiogenin is up-regulated in the nucleus and cytoplasm in human primary breast carcinoma and is associated with markers of hypoxia but not survival. J Pathol.

[67] Larsen AK, Ouaret D, El Ouadrani K, Petitprez A (2011). Targeting EGFR and VEGF(R) pathway cross-talk in tumor survival and angiogenesis. Pharmacol Ther.

[68] Young K, Conley B, Romero D, Tweedie E, O’Neill C, Pinz I, Brogan L, Lindner V, Liaw L, Vary CP (2012). BMP9 regulates endoglin-dependent chemokine responses in endothelial cells. Blood.

[69] Lv LH, Wan YL, Lin Y, Zhang W, Yang M, Li GL, Lin HM, Shang CZ, Chen YJ, Min J (2012). Anticancer drugs cause release of exosomes with heat shock proteins from human hepatocellular carcinoma cells that elicit effective natural killer cell antitumor responses in vitro. J Biol Chem.

[70] Corcoran C, Rani S, O’Brien K, O’Neill A, Prencipe M, Sheikh R, Webb G, McDermott R, Watson W, Crown J, O’Driscoll L (2012). Docetaxel-resistance in prostate cancer: evaluating associated phenotypic changes and potential for resistance transfer via exosomes. PLoS One.

[71] Azmi AS, Bao B, Sarkar FH (2013). Exosomes in cancer development, metastasis, and drug resistance: a comprehensive review. Cancer Metastasis Rev.

[72] Jaiswal R, Luk F, Dalla PV, Grau GE, Bebawy M (2013). Breast cancer-derived microparticles display tissue selectivity in the transfer of resistance proteins to cells. PLoS One.

[73] Press JZ, Reyes M, Pitteri SJ, Pennil C, Garcia R, Goff BA, Hanash SM, Swisher EM (2012). Microparticles from ovarian carcinomas are shed into ascites and promote cell migration. Int J Gynecol Cancer : Off J Int Gynecol Cancer Soc.

[74] Galindo-Hernandez O, Villegas-Comonfort S, Candanedo F, Gonzalez-Vazquez MC, Chavez-Ocana S, Jimenez-Villanueva X, Sierra-Martinez M, Salazar EP (2013). Elevated concentration of microvesicles isolated from peripheral blood in breast cancer patients. Arch Med Res.

[75] Thery C, Amigorena S, Raposo G, Clayton A (2006) Isolation and characterization of exosomes from cell culture supernatants and biological fluids. Curr Protoc Cell Biol Chapter 3:Unit 3 22. doi:10.1002/0471143030.cb0322s30 10.1002/0471143030.cb0322s30 18228490

[76] Touboul C, Lis R, Al Farsi H, Raynaud CM, Warfa M, Althawadi H, Mery E, Mirshahi M, Rafii A (2013). Mesenchymal stem cells enhance ovarian cancer cell infiltration through IL6 secretion in an amniochorionic membrane based 3D model. J Transl Med.

[77] Benelli R, Albini A (1999). In vitro models of angiogenesis: the use of Matrigel. Int J Biol Markers.

