# Supplementary figures and images for: Microparticles mediated cross-talk between tumoral and endothelial cells promote the constitution of a pro-metastatic vascular niche through Arf6 up regulation
Source: Cancer Microenviron. 2014 Jan 15;7(1-2):41–59. doi: 10.1007/s12307-013-0142-2 (PMC4150875; doi:10.1007/s12307-013-0142-2)

Supplementary Figure 1

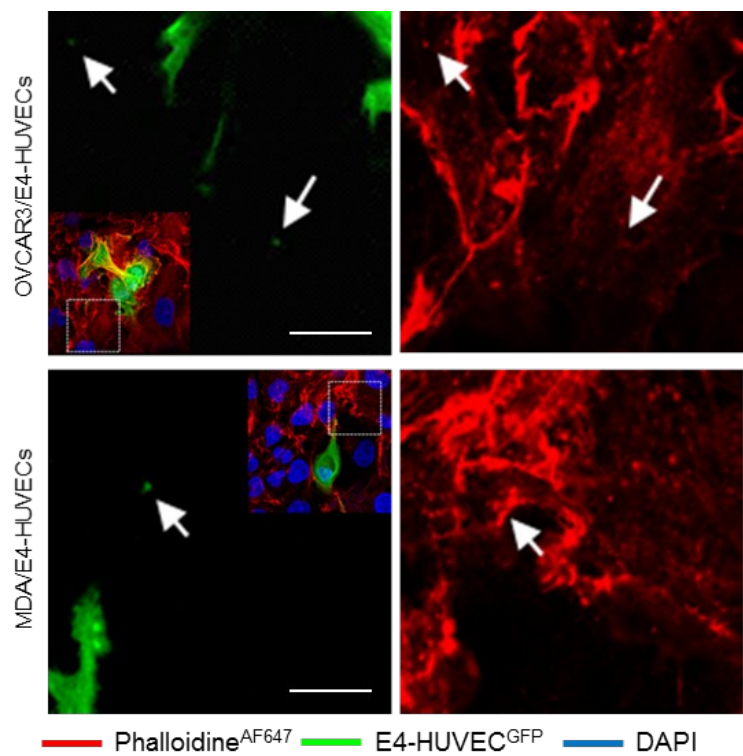

Supplement: Supplementary file 1 — Actin co-localization with MPs. eGFP-E4 + ECs were co-cultured with MDA or OVCAR3 cells for 3 days. Before imaging by confocal microscopy, fixed cells were stained with DAPI and AlexaFluor 647 conjugated-phalloidin. Fixed cells were stained with WGA, DAPI and AlexaFluor 647 conjugated-phalloidin. Arrows indicates area where eGFP-E4 + ECs-MPs (green) co-localize with patches of actin (red). Scale bar 10 μm (PDF 64.5 kb) [file 12307_2013_142_MOESM1_ESM.pdf]

Supplementary Figure 2

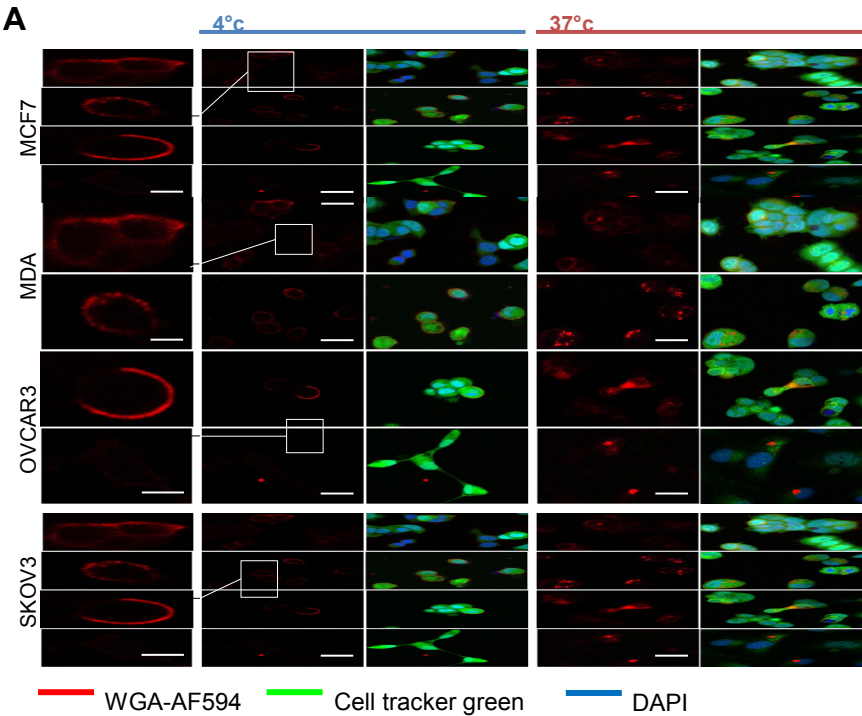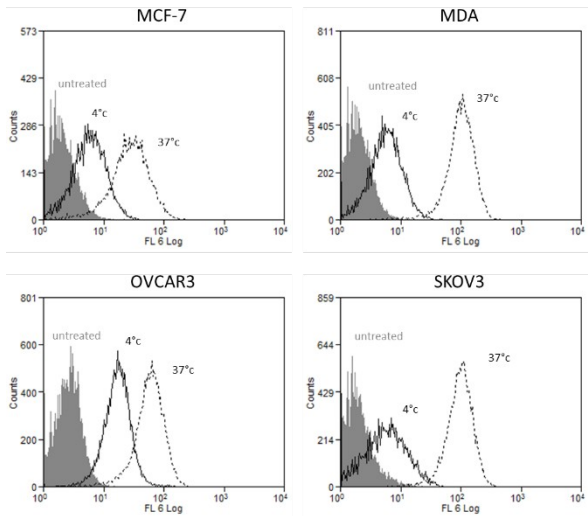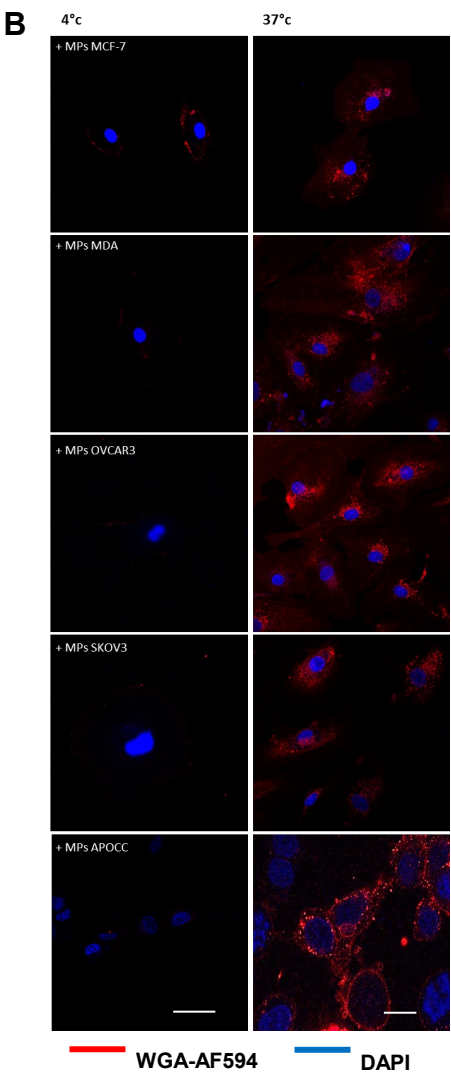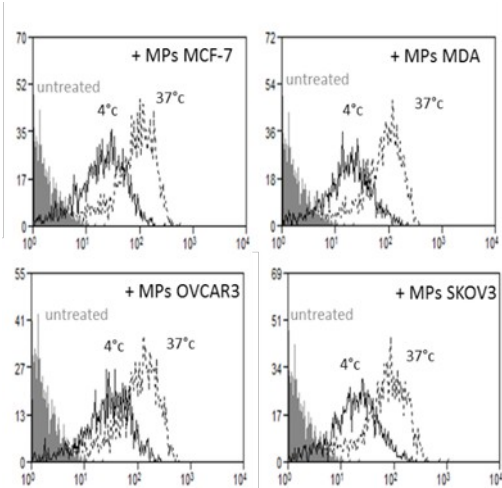

Supplement: Supplementary file 2 — MPs uptake is an active process depending of temperature. A. MPs from E4 + ECs were extracted from 80 % confluent cells and labeled with Alexa Fluor 594 conjugated-wheat germ agglutinin (WGA). Cancer cells lines were incubated with E4 + ECs-MPs for 24 h at 37°c or 4°c. CCs were tagged prior to co-culture with the persistent fluorescent probe CellTracker Green. MPs uptake by the cells only occurs at 37°c. At 4°c, MPs aggregate at the plasma membrane. MPs uptake quantification was performed by flow cytometry (bottom panel). Scale bar 20 μm. B. MPs from all cancer cells lines were extracted from 80 % confluent cells and labeled with Alexa Fluor 594 conjugated-wheat germ agglutinin (WGA). ECs were incubated with the MPs of each cancer cell lines for 24 h at 37°c or 4°c. Quantification of MPs uptake was done by flow cytometry (bottom panel). Scale bar 20 μm. (PDF 392 kb) [file 12307_2013_142_MOESM2_ESM.pdf]

Supplementary Figure 3

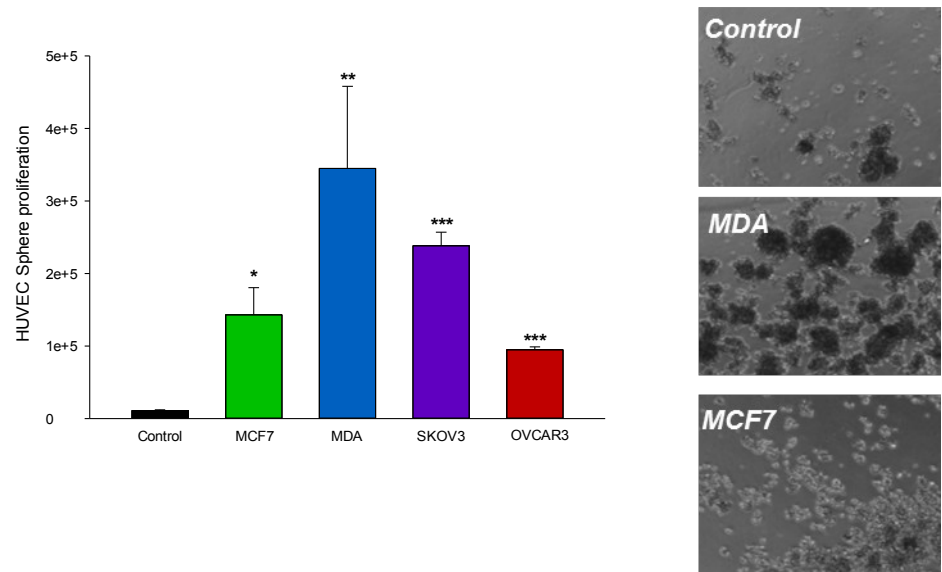

Supplement: Supplementary file 3 — MPs of cancer cell lines increase angiosphere formation. Spheroids of HUVECs were grown in 3D media during 6 days with or without CCs-MPs. Only MPs from MDA and Skov3 sustain the proliferation of HUVECs spheres. (PDF 63.6 kb) [file 12307_2013_142_MOESM3_ESM.pdf]

Supplementary Figure 4

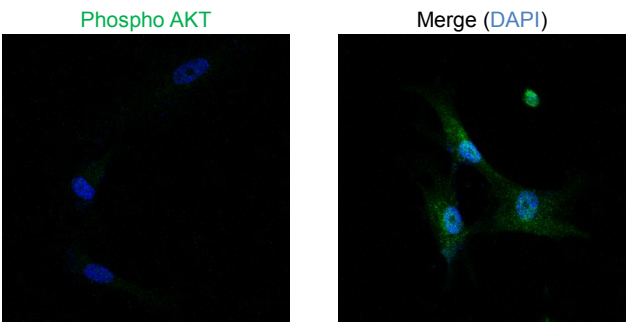

Supplement: Supplementary file 4 — iM-MPs induce Akt phosphorylation in HUVECs. HUVECs were incubated during 30 min with MPs from MCF7 (MCF7-MPs) or MCF7 treated with TGFβ (iM-MPs) and analyzed by confocal microscopy. Only iM-MPs were able to induce phospho-AKT in HUVECs. Scale bar 20 μm. (PDF 50.4 kb) [file 12307_2013_142_MOESM4_ESM.pdf]

Supplementary Figure 5

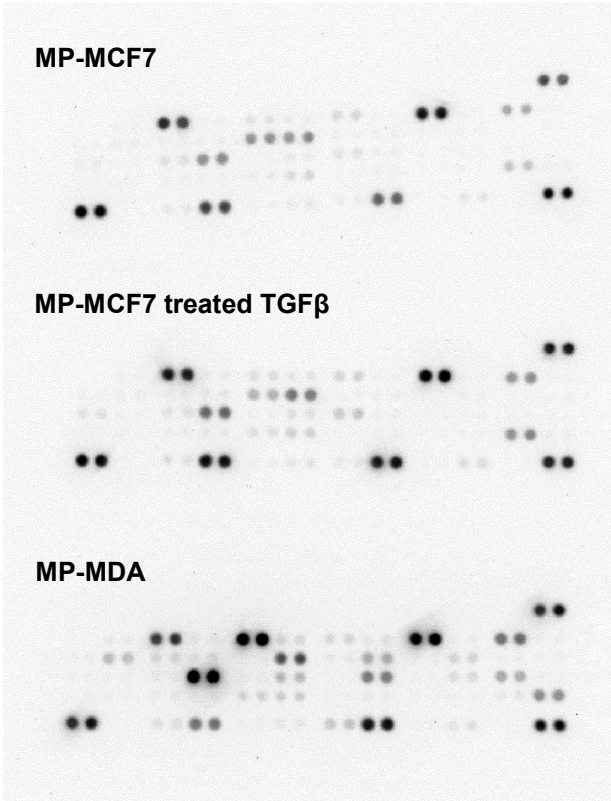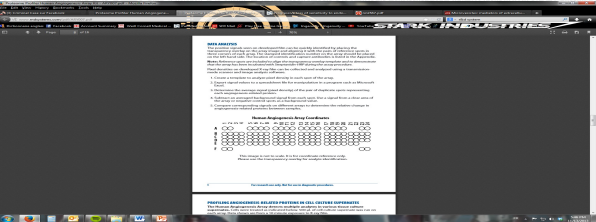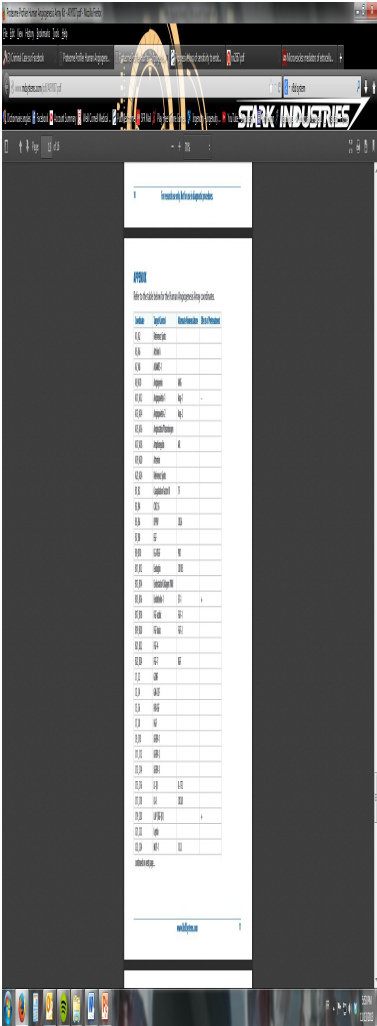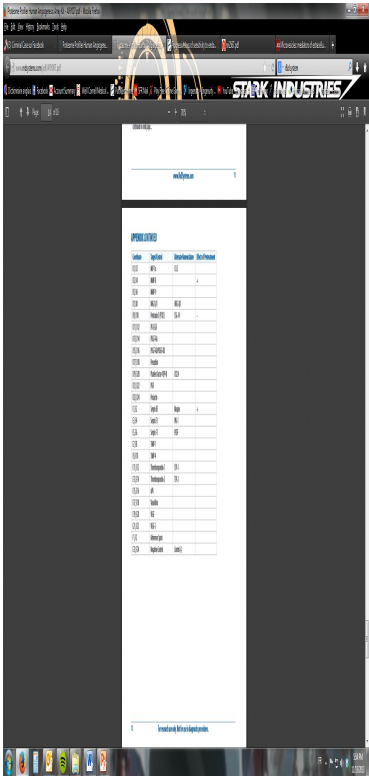

Supplement: Supplementary file 5 — Whole membrane of the Human Angiogenesis Array. Representation of the membrane used for the representation and quantification of angiogenesis-related proteins presented in the figure 5 C. The coordinates and their target were given accordingly to the provider protocol. (PDF 806 kb) [file 12307_2013_142_MOESM5_ESM.pdf]

Supplementary Figure 6

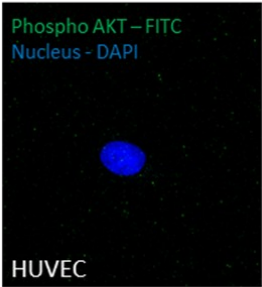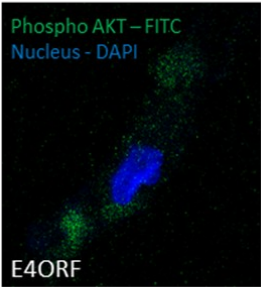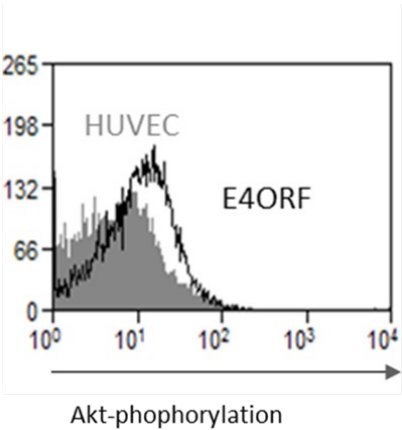

Supplement: Supplementary file 6 — E4 + ECs display an autonomous Akt phosphorylation. Akt phosphorylation level of E4 + ECs in comparison to the HUVECS by confocal microscopy (left panel) and flow cytometry (right panel). (PDF 62.3 kb) [file 12307_2013_142_MOESM6_ESM.pdf]

# Supplementary Figure 7

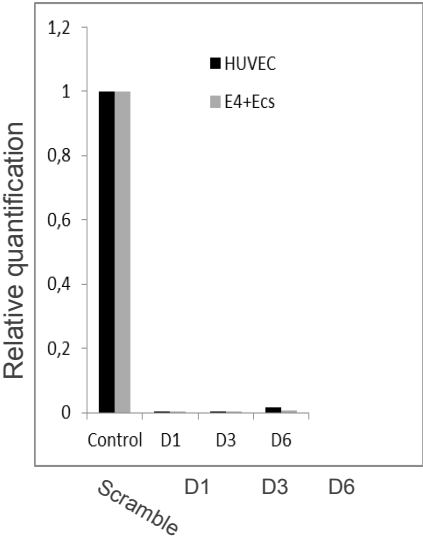

Supplement: Supplementary file 7 — Expression of ARF6 after siRNA treatment. The relative quantification of ARF6 gene was performed by RT-PCR on HUVEC and E4ORF after treatment with SiRNA for ARF6 or the siRNA scramble (control). The ARF6 expression is completely inhibited up to 6 days after the treatment with siRNA. (PDF 33.7 kb) [file 12307_2013_142_MOESM7_ESM.pdf]

Supplementary Figure 8

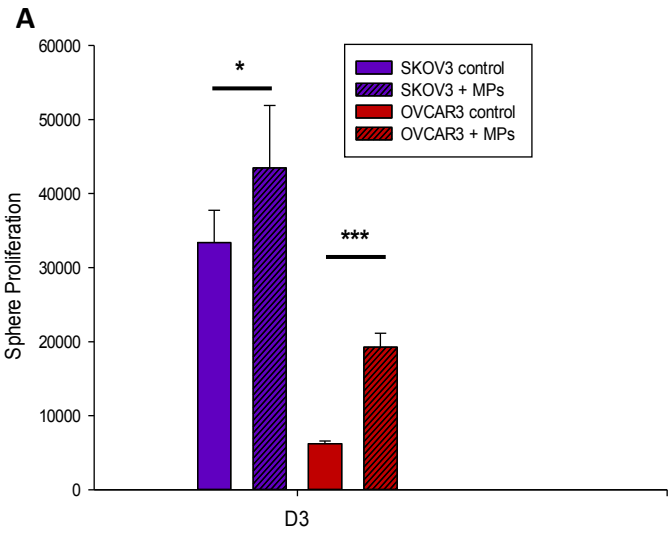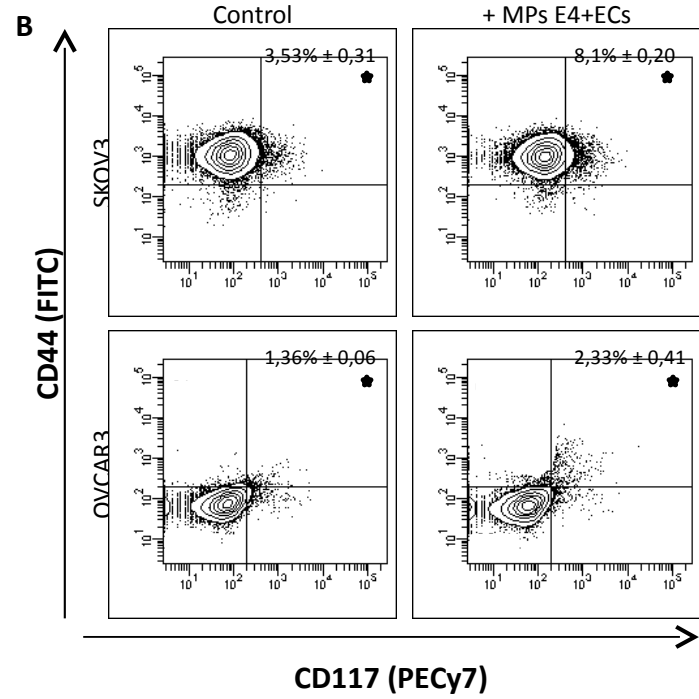

Supplement: Supplementary file 8 — Implication of EC-MPs in cancer stemness. A. Spheroids of CCs were grown in 3D media during 6 days with or without E4 + ECs-MPs. E4 + ECs-MPs sustain the proliferation of CCs spheres. B. CCs were grown with or without E4 + ECs-MPs during 4 days. Before cytometry analysis, ovarian CCs were immunostained with CD44 and CD117. Gate of interest are represented with a star on the graph. E4 + ECs-MPs increase the number of putative cancer stem cells in all CCs population. (PDF 73.4 kb) [file 12307_2013_142_MOESM8_ESM.pdf]
